# Supplementary material for: Potentiators empower synthetic microbiomes as silent guardians against co-contamination
Source: Nat Commun. 2025 Dec 31;17:1185. doi: 10.1038/s41467-025-67953-5 (PMC12858907; doi:10.1038/s41467-025-67953-5)
Supplement: Supplementary file 1 — Supplementary Information [file 41467_2025_67953_MOESM1_ESM.pdf]

Supplementary Information

Supplementary Methods

Supplementary Method 1. Media

1.1 Cultivation Media

The lysogeny broth medium (LB) consisted of 10 g/L tryptone, 10 g/L NaCl, and 5 g/L yeast extract. The composition of the enrichment medium (EM) was 1.5 g/L CH_3COONa, 1.5 g/L NH_4Cl, 1.5 g/L K_2HPO_4, 0.5 g/L KH_2PO_4, 0.2 g/L $MgSO_4 \cdot 7H_2O$, 0.5 g/L NaCl, 1 ml/L trace elements solution, and pH adjusted to 7. The degradation medium (DM) comprised 1.5 g/L peptone, 1.5 g/L NH_4Cl, 1.5 g/L K_2HPO_4, 0.5 g/L KH_2PO_4, 0.2 g/L $MgSO_4 \cdot 7H_2O$, 0.5 g/L NaCl, 1 ml/L trace elements solution, and pH adjusted to 7. The trace elements solution included 0.5 g/L $FeSO_4 \cdot 7H_2O$, 0.5 g/L $MgSO_4 \cdot 7H_2O$, 0.2 g/L $CoCl_2 \cdot 6H_2O$, 0.2 g/L $ZnSO_4$, 0.1 g/L $MnCl_2 \cdot 4H_2O$, 0.5 g/L $CuSO_4 \cdot 5H_2O$, and 1.0 g/L EDTA.

The minimal medium medium (MM) consisted of 1 g/L NaCl, 1 g/L NH_4Cl, 1.5 g/L K_2HPO_4, 0.5 g/L KH_2PO_4, and 0.2 g/L $MgSO_4 \cdot 7H_2O$. The MM-P medium consisted of 10 g/L tryptone, 1 g/L NaCl, 1 g/L NH_4Cl, 1.5 g/L K_2HPO_4, 0.5 g/L KH_2PO_4, and 0.2 g/L $MgSO_4 \cdot 7H_2O$. The MM-10% P medium consisted of 1 g/L tryptone, 1 g/L NaCl, 1 g/L NH_4Cl, 1.5 g/L K_2HPO_4, 0.5 g/L KH_2PO_4, and 0.2 g/L $MgSO_4 \cdot 7H_2O$. The MM-G medium consisted of 10 g/L glucose, 1 g/L NaCl, 1 g/L NH_4Cl, 1.5 g/L K_2HPO_4, 0.5 g/L KH_2PO_4, and 0.2 g/L $MgSO_4 \cdot 7H_2O$.

The LB medium was used for strain activation and large-scale cultivation. The EM medium was used for consortium acclimation, while the DM medium was employed to evaluate the degradation performance of the consortium. The MM medium was used for metabolomic experiments; MM-10% P was used for metabolite supplementation validation experiments; MM-P was used to assess the degradation performance of individual strains and to verify the loss-of-function of enhancer strains. The MM-G medium was used to determine the growth curves of the top 50 strains from the TCs subset.

1.2 Modeling Media

For single-strain models:

First, the medium was configured according to the exchange metabolites included in each single-strain metabolic model. Among the components, the minimal carbon source (e.g., glucose) and NH_4^+ were set with an LB of 100 mmol/gDW; all other organic substances were set with an LB of 0 mmol/gDW; and all other inorganic substances without carbon (C) or nitrogen (N) were set to the maximum value (an LB of 1000 mmol/gDW). The UB for all substances was uniformly set to 1000 mmol/gDW. Through this configuration, the minimal medium for each single strain was determined. Notably, in the single-strain medium, the LB of CO_2 was set to 0 mmol/gDW, and the UB of O_2 was set to 0 mmol/gDW.

For community models:

The medium was the union of the minimal media of all individual strains in the community. Similar to the single-strain setup, only the target carbon sources were specifically configured (e.g., configurations for C = 1 and C = 4 conditions), with details as follows:

- C = 1 condition: 100 mmol glucose ($C_6H_{12}O_6$, containing 6 carbon atoms per mole) was used as the sole carbon source, resulting in a total carbon input of 100 mmol × 6 = 600 mmol C.
- C = 4 condition: Four carbon sources were included, each at 25 mmol to ensure balanced representation: 25 mmol glucose (6 C atoms, contributing 150 mmol C), 25 mmol citrate (6 C atoms, contributing 150 mmol C), 25 mmol acetate (2 C atoms, contributing 50 mmol C), and 25 mmol fumarate (4 C atoms, contributing 100 mmol C). The total carbon input (for the C = 4 condition) was 150 + 150 + 50 + 100 = 450 mmol C.

While NH_4^+ still had an LB of 100 mmol/gDW, all other organic substances had an LB of 0 mmol/gDW, and all other inorganic substances without C/N had an LB of 1000 mmol/gDW. The UB for all substances was consistently set to 1000 mmol/gDW, thereby determining the minimal medium for the microbial community. Additionally, the LB of CO_2 (0 mmol/gDW) and UB of O_2 (0 mmol/gDW) were maintained the same as in the single-strain medium.

Supplementary Method 2. HPLC and LC-MS analysis

2.1 Quantification of tetracycline and oxytetracycline by means of LC-MS

We conducted a quantitative analysis of tetracycline (TC), oxytetracycline (OTC), dodecyl trimethyl ammonium chloride (DTAC), dodecyldimethylbenzylammonium chloride (DBAC), sulfamethoxazole (SMX), and sulfamethazine (SMT) using a liquid chromatography-mass spectrometry (LC-MS) system (Agilent 6470B). Chromatographic separation was achieved on a C_{18} reversed-phase column (150 mm × 4.6 mm, 5 μm particle size). For the determination of TC, OTC, DTAC, DBAC, SMX, and SMT, 1 mL of the water sample was transferred into a 1.5-mL centrifuge tube and centrifuged at 6000 rpm for 10 min. The supernatant was collected and used directly for LC–MS analysis. For BO, 1 mL of the water sample was transferred into a 10-mL centrifuge tube, followed by the addition of an equal volume of dichloromethane. After vortexing for 10 min, the mixture was allowed to settle for phase separation. The aqueous phase was discarded, and the organic phase was collected and evaporated to dryness under a nitrogen stream. The residue was then reconstituted in 1 mL of methanol prior to LC–MS injection. All samples were analyzed in triplicate. LC–MS data acquisition was performed using Agilent MassHunter (version B.10.1.67).

For TC and OTC, the injection volume was 2 μL. Mobile phase A consisted of 0.1 % formic acid in water, prepared using a Milli-Q Advantage ultrapure water system, while mobile phase B was HPLC-grade acetonitrile (purchased from Shanghai Anpu Co., Ltd.). The flow rate was maintained at 0.3 mL/min. The elution gradient was as follows: 0-5 min, 90 % to 60 % mobile phase A; 5-5.1 min, 60 % to 90 % mobile phase A; and 5.1-7.1 min, 90 % mobile phase A. The column temperature was set at 30 °C. Mass spectrometry was performed using an electrospray ionization (ESI) source with Agilent Jet Stream technology in positive ion mode. The scan type was set to multiple reaction monitoring (MRM) with a Delta EMV (+) of 300. The MRM parameters for TC were as follows: precursor ion 445.1 m/z, quantifier ion 410.1 m/z, qualifier ion 154 m/z, with a fragmentation voltage of 150 V. Collision energy (CE) for ion 410.1 was set to 21 V, and for 154 to 29 V, with a cell acceleration voltage of 4 V and positive polarity. For OTC, the precursor ion was 461.2 m/z, quantifier ion 426.1 m/z, and qualifier ion 443.1 m/z, with a fragmentation voltage of 125 V and CE of 21 V and 13 V. The cell acceleration voltage was 4 V, also in positive polarity. The retention times

for TC and OTC were 2.6 min and 2.4 min, respectively. The limits of detection (LOD) were 0.12383 ppb for TC and 8.47458 ppb for OTC.

For DTAC and DBAC, the injection volume was 5 μL. Mobile phase A was 0.1 % formic acid in water, and mobile phase B was acetonitrile. The flow rate was 0.3 mL/min. The elution gradient was: 0-1.5 min, 55 % to 20 % mobile phase A; 1.5-3 min, 20 % to 0 % mobile phase A; 3-6 min, 0% mobile phase A; and 6-10 min, 55 % mobile phase A. The column temperature was 40 °C. The MRM parameters for DBAC were as follows: precursor ion 304.3 m/z, quantifier ion 91.2 m/z, qualifier ion 212.2 m/z, with a fragmentation voltage of 125 V. CE for ion 91.2 was set to 40 V, and for 212.2 to 20 V, with a cell acceleration voltage of 4 V and positive polarity. For DTAC, the precursor ion was 228.3 m/z, quantifier ion 60.4 m/z, and qualifier ion 71.3 m/z, with a fragmentation voltage of 125 V and CE of 24 V and 28 V. The cell acceleration voltage was 4 V, also in positive polarity. The retention times for DBAC and DTAC were 2.1 min and 1.6 min, respectively. The LOD values were 0.00739 ppb for DBAC and 0.01044 ppb for DTAC.

For SMX and SMT, the injection volume was 2 μL. Mobile phase A was 0.1 % formic acid in water, and mobile phase B was acetonitrile. The flow rate was 0.3 mL/min. The elution gradient was: 0-0.5 min, 90 % mobile phase A; 0.5-1 min, 90 % to 10% mobile phase A; 1-2.5 min, 10 % mobile phase A; and 2.5-3 min, 10 % to 90 % mobile phase A. The column temperature was 35 °C. The MRM parameters for SMT were as follows: precursor ion 279 m/z, quantifier ion 186 m/z, qualifier ion 92.1 m/z, with a fragmentation voltage of 109 V. CE for ion 186 was set to 17 V, and for 92.1 to 35 V, with a cell acceleration voltage of 4 V and positive polarity. For SMX, the precursor ion was 254 m/z, quantifier ion 92 m/z, and qualifier ion 156 m/z, with a fragmentation voltage of 109 V and CE of 32 V and 16 V. The cell acceleration voltage was 4 V, also in positive polarity. The LOD values were 0.02244 ppb for SMX and 0.00431 ppb for SMT.

For BO, the injection volume was 2 μL. Mobile phase A was 0.5 % acetic acid in water, and mobile phase B was acetonitrile. The flow rate was 0.3 mL/min. The elution gradient was: 0-2 min, 20% mobile phase A. The column temperature was 30 °C. The MRM parameters for BO were as follows: precursor ion 403 m/z, quantifier ion 300 m/z, qualifier ion 344 m/z, with a fragmentation voltage of 195 V. CE for ion 300 was set to 32 V, and for 344 to 28 V, with a cell acceleration voltage

of 4 V and positive polarity. The LOD for BO was 0.0728 ppb.

2.2 Quantification of pollutants by means of HPLC

The analysis of bromoxynil octanoate (BO) and quinclorac (QC) was performed using a high-performance liquid chromatography (HPLC) system (Agilent 1260). Detection of BO was facilitated through its intermediate metabolite, bromoxynil (Bxn).

For the chromatographic separation of Bxn, a C_{18} reversed-phase column (150 mm × 4.6 mm, 4 μm particle size) was employed. Bxn was detected at a wavelength of 250 nm. The column temperature was maintained at 40 °C, and the injection volume was set to 20 μL. The mobile phase consisted of a mixture of acetonitrile/water/acetic acid (50/49.5/0.5, v/v/v), with a flow rate of 1.0 mL/min. The chromatographic separation of quinclorac was achieved using a C_{18} reversed-phase column (250 mm × 4.6 mm, 5 μm particle size). QC was detected at a wavelength of 240 nm. The column temperature was maintained at 30 °C, and the injection volume was set to 10 μL. The mobile phase for quinclorac was a mixture of methanol/water/phosphoric acid (75/24.8/0.2, v/v/v), with a flow rate of 1.0 mL/min. The retention times for Bxn and quinclorac were 3.5 minutes and 3.9 minutes. The LOD were 180 ppb for Bxn and 6.6 ppb for QC.

Supplementary Method 3. Metabolomic analysis

After 4 hours of cultivation, the entire culture was centrifuged at 6000 rpm for 10 minutes. The supernatant was transferred to sterile microcentrifuge tubes, frozen at −80 °C, and lyophilized for further analysis. For metabolite extraction, 50 mg of the freeze-dried sample and a 6 mm diameter grinding bead were added to a 2 mL centrifuge tube. Then, 400 μL of extraction solution (methanol:water 4:1, v/v, containing 0.02 mg/mL L-2-chlorophenylalanine as an internal standard) was added. The samples were ground using a Wonbio-96c frozen tissue grinder at −10 °C and 50 Hz for 6 min, followed by sonication at 5 °C and 40 kHz for 30 min. After incubation at −20 °C for 30 min, the samples were centrifuged at 4 °C and 13000 g for 15 min, and the supernatant was transferred to an injection vial.

Liquid chromatography-tandem mass spectrometry (LC-MS/MS) analysis was performed using

a Thermo UHPLC-Q Exactive HF-X system (Thermo Fisher Scientific) equipped with an ACQUITY HSS T3 column (100 mm × 2.1 mm i.d., 1.8 μm; Waters, USA) at Majorbio Bio-Pharm Technology Co. Ltd. (Shanghai, China). The mobile phases consisted of solvent A (0.1 % formic acid in water:acetonitrile, 95:5, v/v) and solvent B (0.1 % formic acid in acetonitrile:isopropanol:water, 47.5:47.5:5, v/v). The flow rate was maintained at 0.40 mL/min, and the column temperature was set at 40 °C. The injection volume was 3 μL. Mass spectrometric data were acquired using a Thermo UHPLC-Q Exactive HF-X Mass Spectrometer with an electrospray ionization (ESI) source in both positive and negative ion modes. The optimized conditions were as follows: auxiliary gas heating temperature, 425 °C; capillary temperature, 325 °C; sheath gas flow rate, 50 psi; auxiliary gas flow rate, 13 psi; ion-spray voltage floating (ISVF), −3500 V (negative mode) and 3500 V (positive mode). The normalized collision energy was set to 20, 40, and 60 eV for MS/MS. The full MS resolution was 60,000, and the MS/MS resolution was 7500. Data acquisition was conducted in Data Dependent Acquisition (DDA) mode with a mass range of 70–1050 m/z.

The UHPLC-MS raw data were processed for baseline filtering, peak identification, peak integration, retention time correction, and peak alignment. The resulting data matrix, containing sample names, m/z values, retention times, and peak intensities, was exported for further analysis. Metabolites were identified by searching against the Human Metabolome Database (HMDB, http://www.hmdb.ca/), METLIN (https://metlin.scripps.edu/), and the self-compiled Majorbio Database (MJDB) from Majorbio Biotechnology Co., Ltd. (Shanghai, China). The data matrix was uploaded to the Majorbio Cloud Platform (https://cloud.majorbio.com) for analysis. Data preprocessing included retaining variables detected in at least 80 % of samples, imputing missing values with the minimum value in the matrix, and normalizing peak intensities using the sum normalization method.

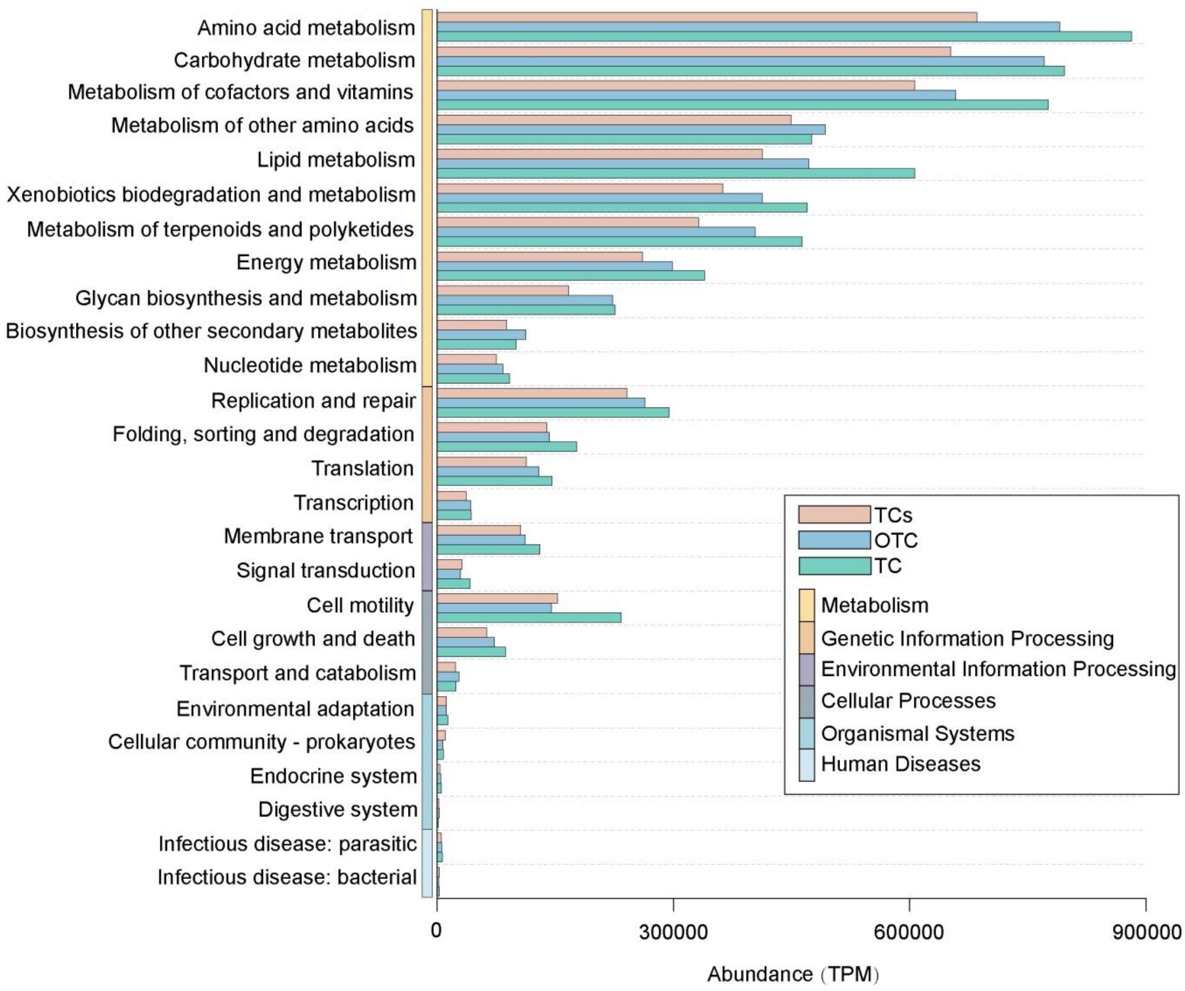

Supplementary Fig. 1 | KEGG annotation of level 1 and level 2 of acclimated microbial consortia. TC, tetracycline; OTC, oxytetracycline; TCs, TC&OTC. The data are presented as mean values (n = 3 biological independent replicates). Source data for this figure is available in the Source Data file.

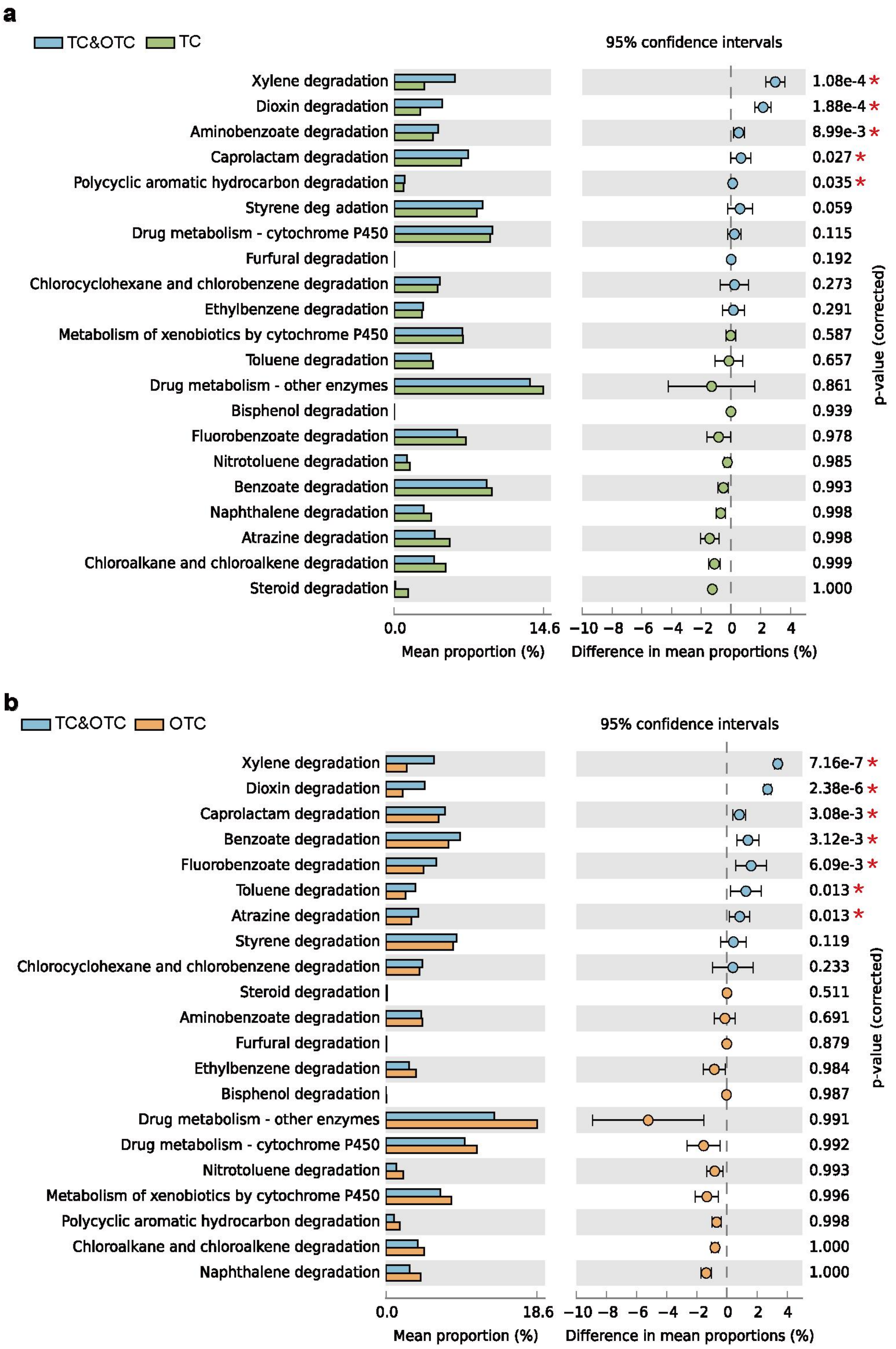

Supplementary Fig. 2 | Functional divergence of acclimated microbial consortia. a, STAMP analysis of xenobiotic biodegradation genes showing differential abundance between TC- and TC&OTC-acclimated consortia. **b**, Comparative STAMP analysis of xenobiotic degradation genes between OTC- and TC&OTC-acclimated consortia. A one-tailed t-test with a 0.95 confidence level was employed to assess the equality of variances. The data are presented as mean values (n = 3 biological independent replicates). TC, tetracycline; OTC, oxytetracycline. *, p < 0.05. Source data for this figure is available in the Source Data file.

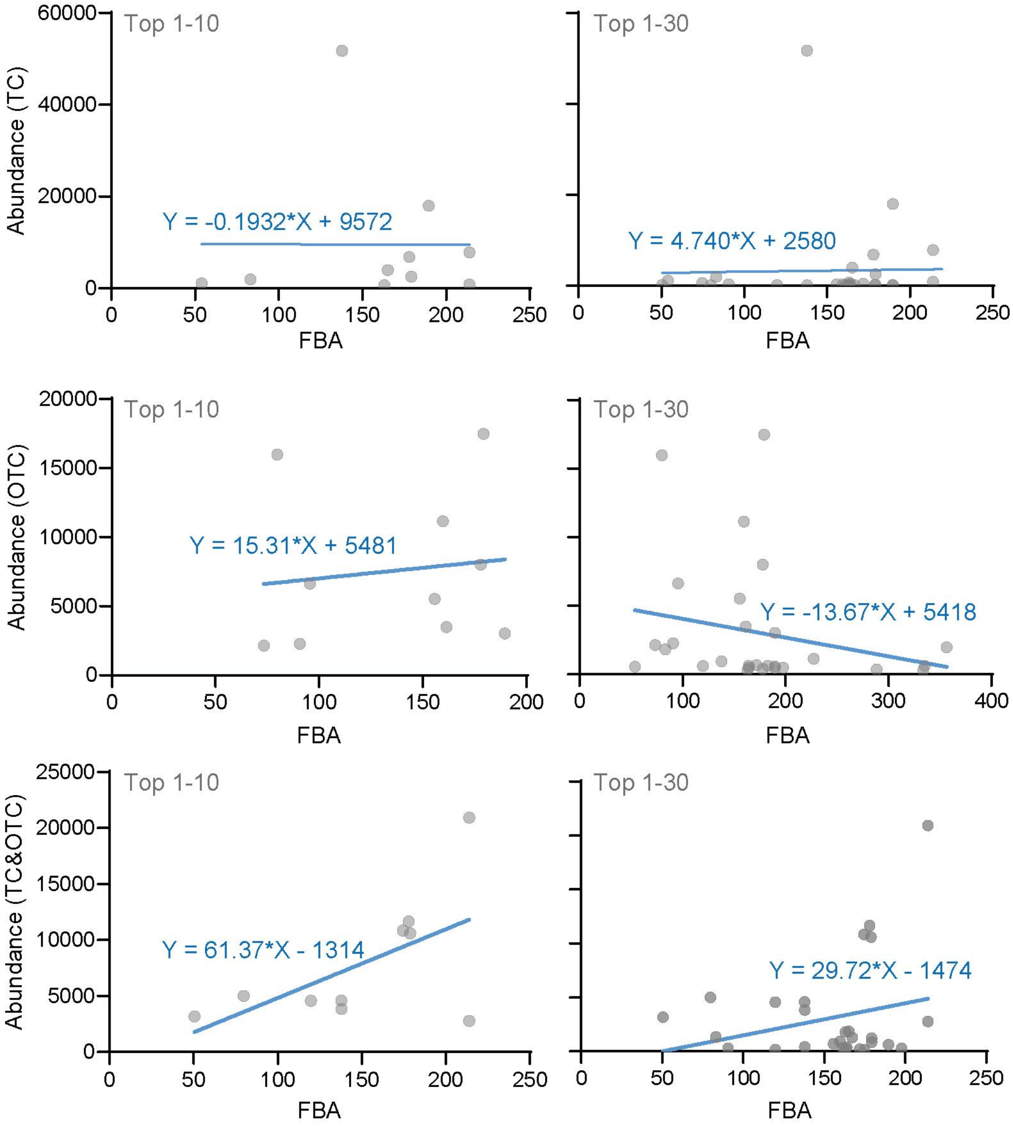

Supplementary Fig. 3 | Correlation analysis between the abundances of Top 1-10 and Top 1-30 species and their maximum biomass under optimal conditions (simulated by FBA) across three treatments: TC, OTC, and TC&OTC. TC, tetracycline; OTC, oxytetracycline. CK, control group. The data of the abundances are presented as mean values. Source data for this figure is available in the Source Data file.

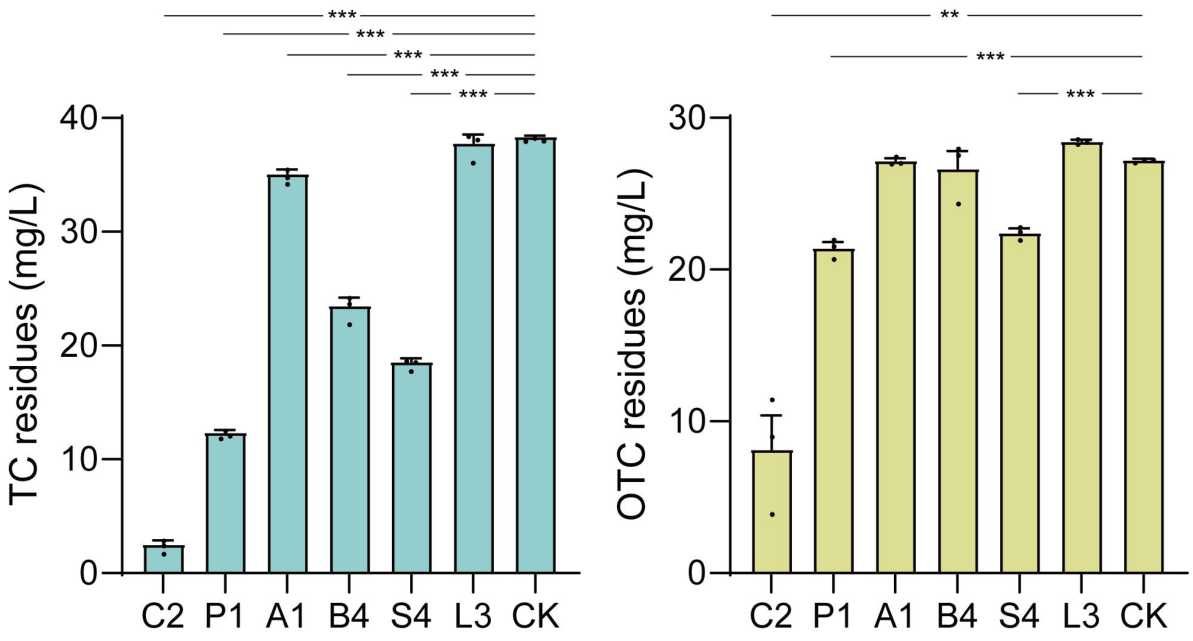

Supplementary Fig. 4 | Degradation capacity of six keystone strains against TC and OTC. Since none of these strains could utilize the pollutants as sole carbon sources, degradation tests were conducted in minimal medium (MM) supplemented with peptone, containing either 30 mg/L TC or OTC for 3 days' cultivation. *, $p < 0.05$; **, $p < 0.01$; ***, $p < 0.001$. TC, tetracycline; OTC, oxytetracycline. CK, control group. The data are presented as mean values ± SD (n = 3 biological independent replicates). Source data for this figure is available in the Source Data file.

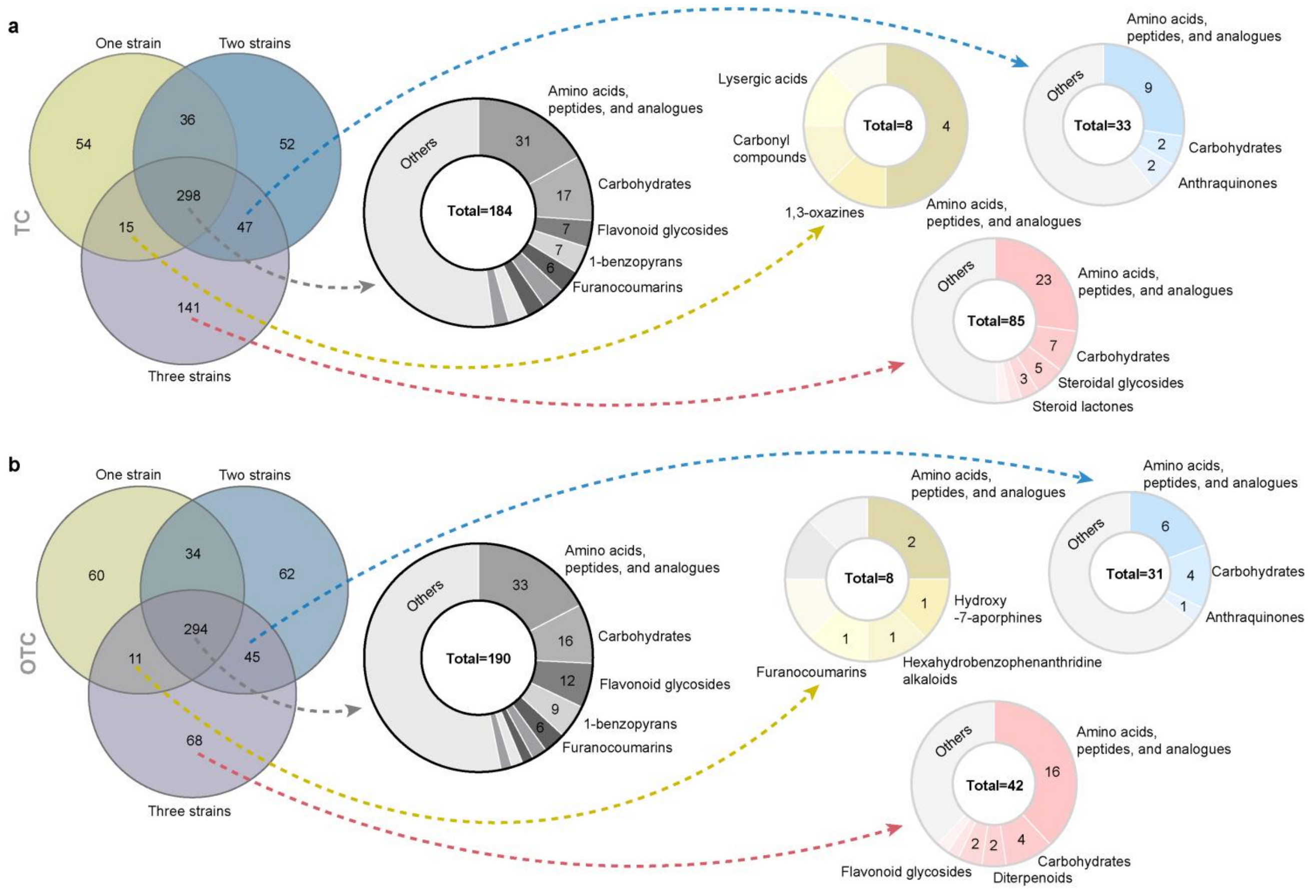

Supplementary Fig. 5 | Differential metabolite analysis in TC/OTC groups. a, Metabolite analysis in TC group. **b**, Metabolite analysis in OTC group. Venn diagram shows significantly upregulated metabolites (vs CK; $p < 0.01$, VIP ≥ 2). Circular plots display HMDB annotation classes. The numbers in the middle of the circle diagram represent the number of metabolites that have been annotated. TC, tetracycline; OTC, oxytetracycline. The data are presented as mean values (n = 4 biological independent replicates). Source data for this figure is available in the Source Data file.

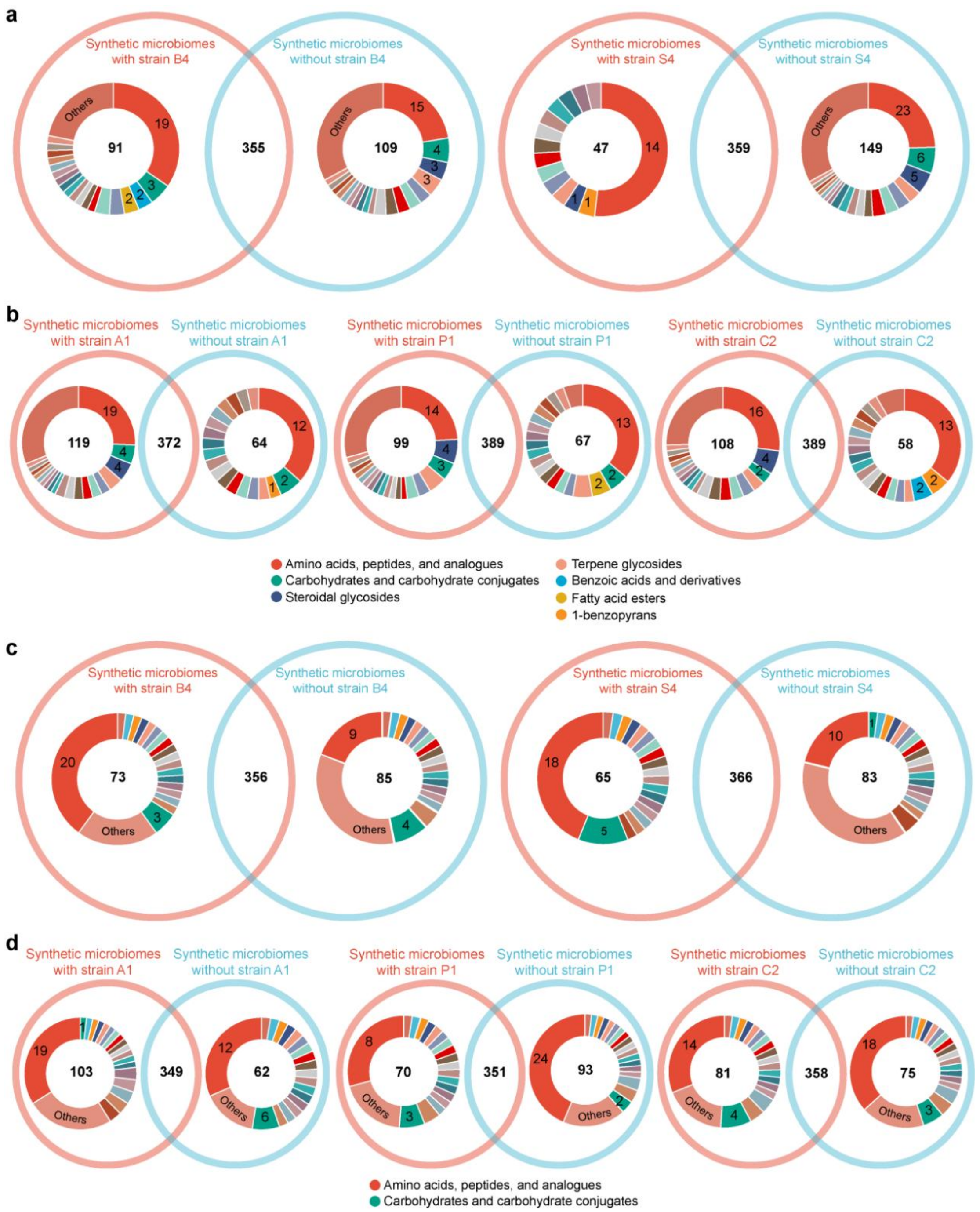

Supplementary Fig. 6 | Venn diagram and functional annotation of metabolites. **a**, The differences in microbial metabolites of TCs-group with or without strain B4/S4 in the synthetic microbiomes. **b**, The differences in microbial metabolites of TCs-group with or without strain A1/P1/C2 in the synthetic microbiomes. **c**, The differences in microbial metabolites of OTC-group with or without strain B4/S4 in the synthetic microbiomes. **d**, The differences in microbial metabolites of OTC-group with or without strain A1/P1/C2 in the synthetic microbiomes. Circular sectors display HMDB-annotated metabolite classes. TCs, tetracycline&oxytetracycline (vs CK; $p < 0.05$, VIP $\geqslant$ 1). Source data for this figure is available in the Source Data file.

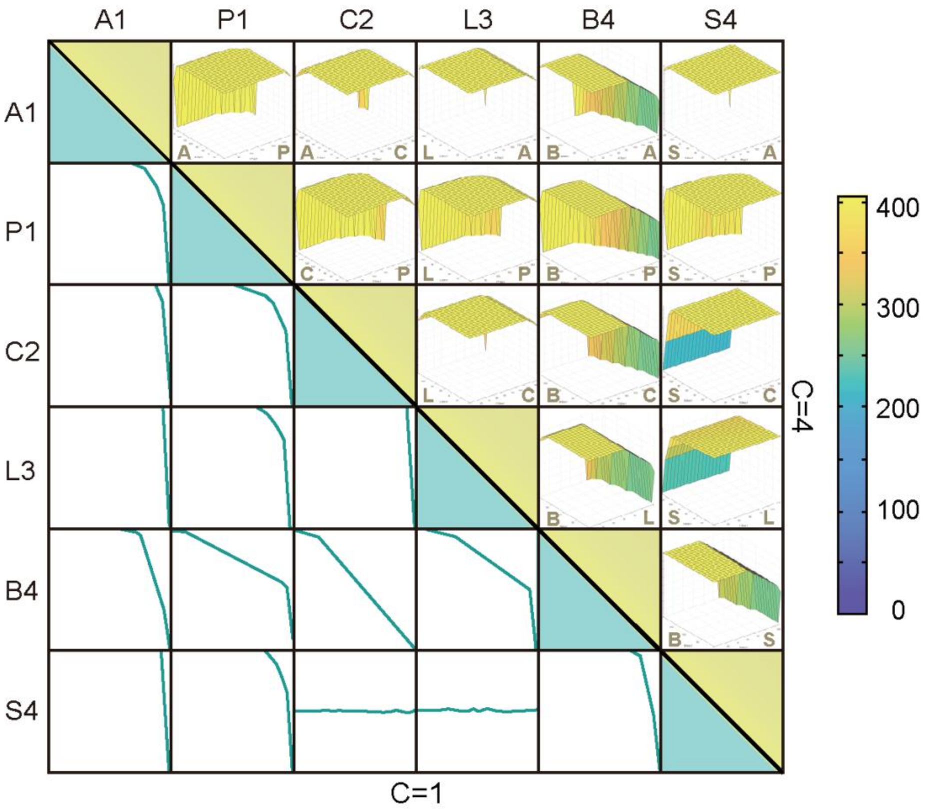

Supplementary Fig. 7 | Simulation of Metabolic Interactions between Paired Strains. The lower left part, unidirectional coupling analysis (C = 1). When optimizing biomass production of a single target strain (objective function), the dependent biomass variation of its partner strain was computationally resolved. The upper right part, bidirectional co-growth simulation (C = 4). Under multi-nutrient conditions, simultaneous biomass optimization of both strains was performed, with total consortium biomass calculated across their combined growth space. C = 1, MM + 100 mmol/gDW glucose (single carbon source); C = 4, MM + 25 mmol/gDW each of glucose, citrate, acetate, and fumarate (mixed carbon sources). The source code used to generate this figure is available in the SuperCC metabolic modeling pipeline repository at: https://github.com/ruanzhepu/superCC.git.

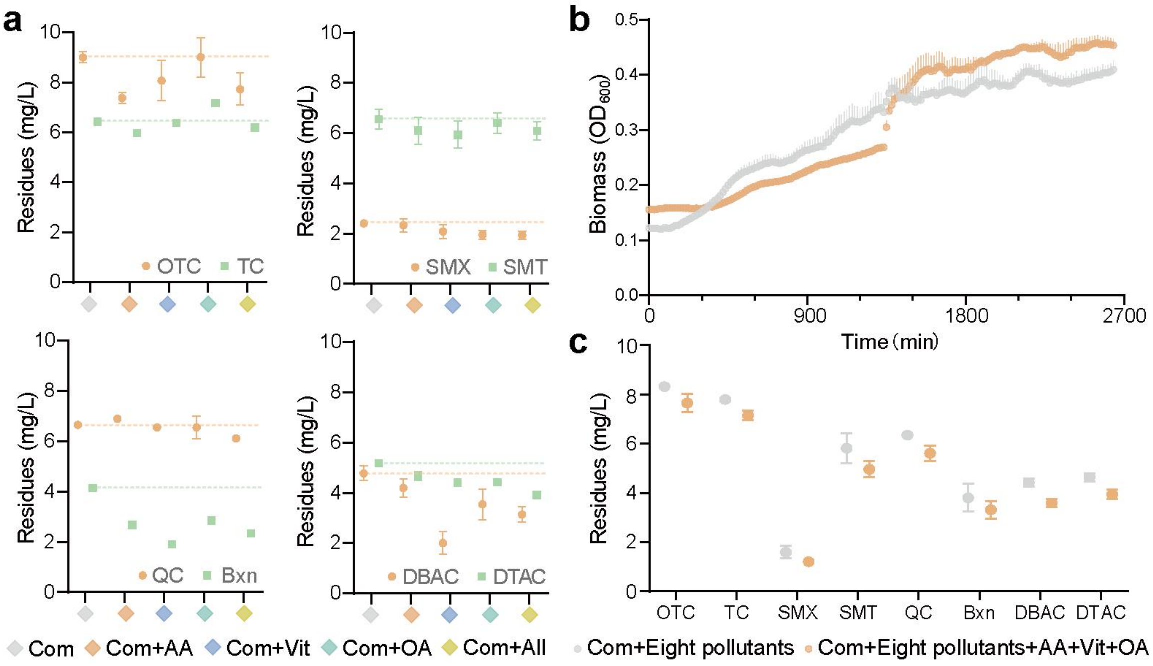

Supplementary Fig. 8 | Experimental validation of predicted cross-talk metabolites under eight co-contaminated pollutants. **a**, Enhanced degradation efficiency. The pollution residues demonstrate the response of microbial communities to four types of combined pollution (10 mg/L each), including: tetracyclines (TC and OTC), sulfonamide antibiotics (SMX and SMT), herbicides (BO and QC), and biocides (DTAC and DBAC). The cultures were supplemented with different metabolite combinations and monitored every 15 min: the control group (Com) contained only the basal medium (10% peptone), while experimental groups received additional amino acids (Com+AA: L-histidine, L-glutamate, L-phenylalanine, N-formyl-L-methionine, glycine, and L-proline), vitamins (Com+Vit: vitamin B1, vitamin D2, and niacin), organic acids (Com+OA: palmitate, myristic acid, and succinate), or all key metabolites combined (Com+All). The degradation performance across different pollution categories shows the improvement conferred by metabolite supplementation. Dashed lines represent the residual pollutant levels in the unsupplemented control (Com) for comparison. **b**, Growth promotion by metabolite supplementation. The growth curves demonstrate the response of microbial communities to four types of combined pollution (10 mg/L each). The cultures were supplemented with different metabolite combinations and monitored every 15 min: the control group (Com+Eight pollutants) contained only the basal medium (10% peptone) and pollutants, while experimental groups received additional amino acids, vitamins, and organic acids. The data are presented as mean values ± SD (n = 4 biological independent replicates). **c**, Enhanced degradation efficiency. The degradation performance across different pollution categories shows the improvement conferred by metabolite supplementation. The x-axis indicates treatment groups using the same color scheme as (**b**). The data are presented as mean values ± SD (n = 3 biological independent replicates). AA, amino acid; Vit, vitamin; OA, organic acid; TC, tetracycline; OTC, oxytetracycline; DTAC, dodecyl trimethyl ammonium chloride; DBAC, dodecyldimethylbenzylammonium chloride; BO, bromoxynil octanoate; Bxn, bromoxynil (intermediate metabolite of BO); QC, quinclorac; SMX, sulfamethoxazole; SMT, sulfamethazine. Source data for this figure is available in the Source Data file.

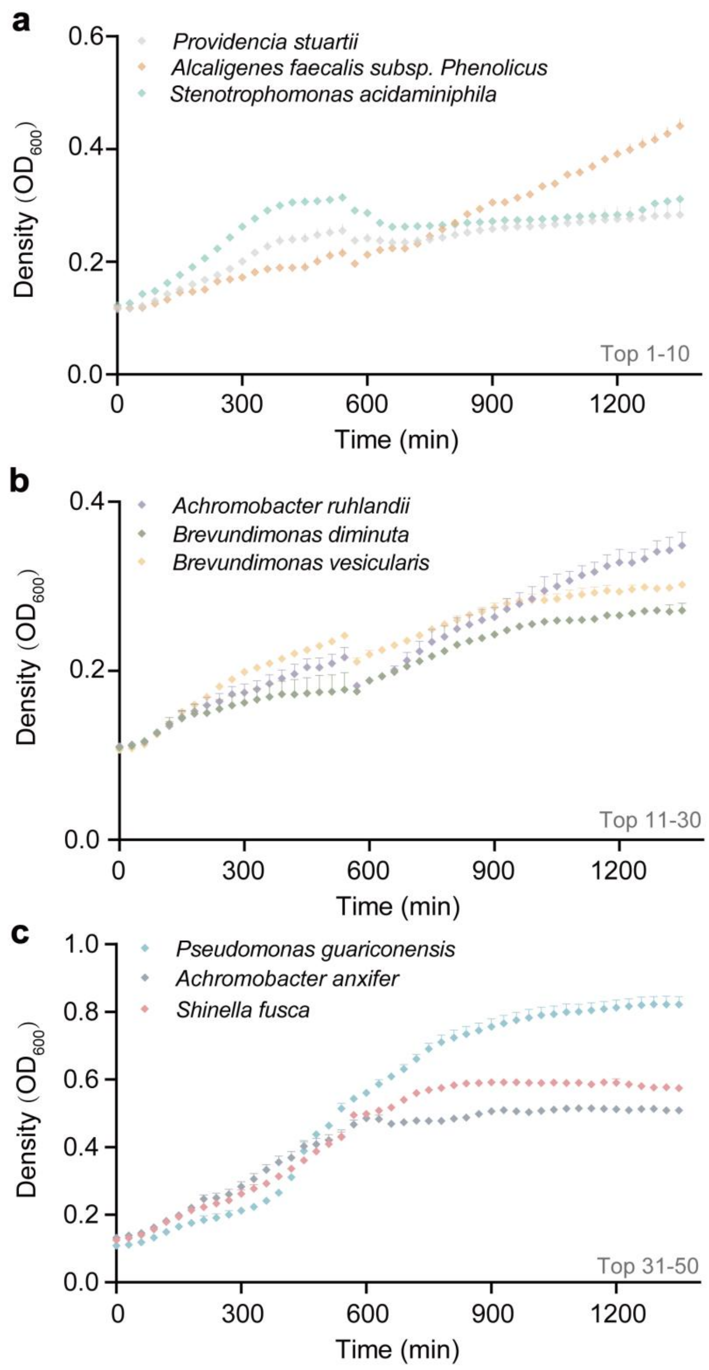

Supplementary Fig. 9 | Growth curves of representative species from the co-contamination of the top 50 keystone strains. Bacterial growth curves were determined in an inorganic salt medium supplemented with 1 mol/L glucose (MM-G). The strains were divided into three subsets based on their predicted ranking: **a**, growth curves of three representative strains from the Top 1-10 subset; **b**, growth curves of three representative strains from the Top 11-30 subset; **c**, growth curves of three representative strains from the Top 31-50 subset. The data are presented as mean values (n = 4 biological independent replicates). Source data for this figure is available in the Source Data file.

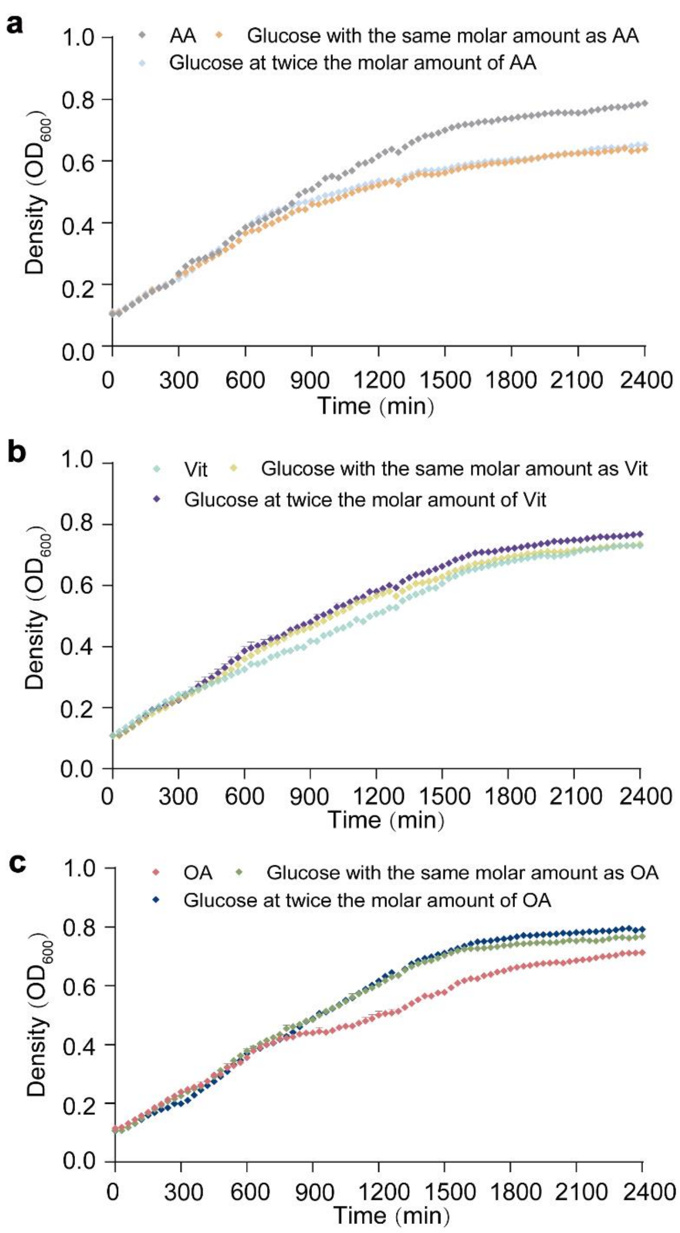

Supplementary Fig. 10 | Equivalent glucose supplementation experiments. Control experiments were conducted to assess whether the growth-promoting effects of metabolite supplementation were attributable to additional carbon input rather than the specific metabolites themselves. Growth curves of microbial consortia under TC and OTC co-contamination with supplementation of (**a**) amino acids (AA), (**b**) vitamins (Vit), or (**c**) organic acids (OA) were compared with glucose supplementation providing an equivalent or double molar amount of carbon. Each panel shows three treatments: addition of the corresponding metabolite (AA, Vit, or OA), glucose at the same molar amount of carbon as the metabolite, and glucose at twice the molar amount of carbon. Growth was monitored by optical density at 600 nm over 2400 min. TC, tetracycline; OTC, oxytetracycline. The data are presented as mean values (n = 4 biological independent replicates). Source data for this figure is available in the Source Data file.

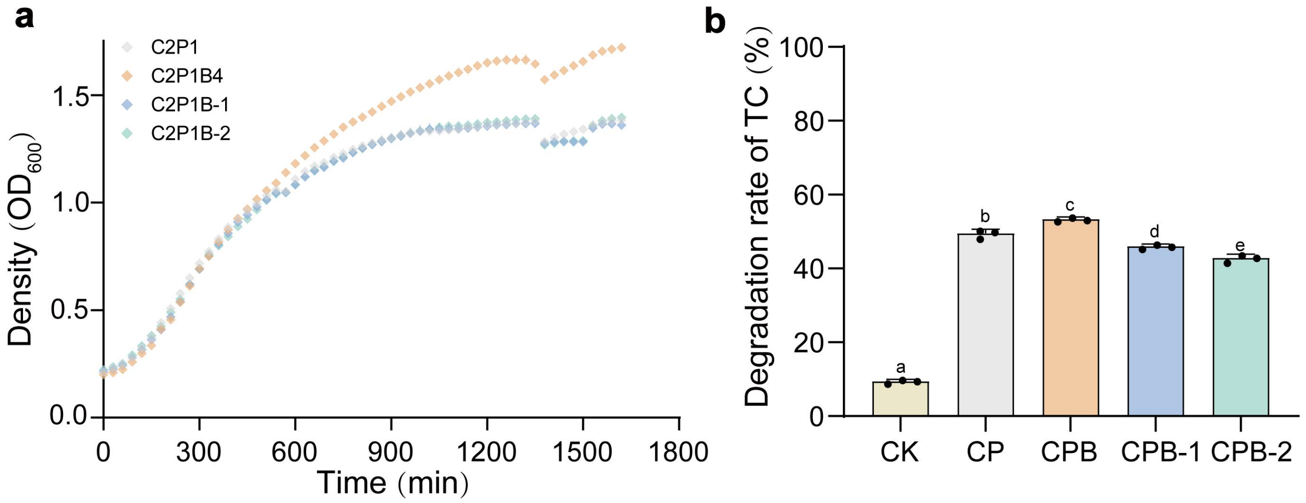

Supplementary Fig. 11 | Community member substitution test. Different bacterial consortia were inoculated into MM-peptone medium containing 10 ppm TC and OTC and incubated for 27 h: CP (the combination of strains *Comamonas* sp. C2 and *Providencia* sp. P1), CPB (the combination of strains *Comamonas* sp. C2, *Providencia* sp. P1, and *Brevundimonas* sp. B4), CPB-1 (the combination of strains *Comamonas* sp. C2, *Alcaligenes* sp. A1, and *Brevundimonas vesicularis*, a strain of the same genus but different species from B4), and CPB-2 (the combination of strains *Comamonas* sp. C2, *Providencia* sp. P1, and *Caulobacter vibrioides*, a bacterium from the same family but different genus from B4). The optical density and degradation rate of TC were measured after 27 h of cultivation. **a**, Growth curves of different consortia. **b**, Degradation rates of TC different consortia. TC, tetracycline; OTC, oxytetracycline. The data are presented as mean values (n = 4 biological independent replicates). Source data for this figure is available in the Source Data file.

Supplementary Table 1 Keystone isolated strains used in the study and general features of the metabolic models constructed for these strains.

Strains	Genus	ASV	Genome accession*	Reactions	Exchange reactions	Transport reactions	Biochemical reactions	Metabolites
**A1**	*Alcaligenes*	ASV39	CP190004	1456	115	121	1220	1520
**P1**	*Providencia*	ASV9	CP190008	1536	107	116	1313	1508
**C2**	*Comamonas*	ASV1	CP190002-CP190003	1478	82	92	1304	1588
**L3**	*Leucobacter*	ASV50	CP190007	1255	89	94	1072	1295
**B4**	*Brevundimonas*	ASV2	CP190005	1272	80	91	1101	1324
**S4**	*Stenotrophomonas*	ASV15	CP190006	1350	73	66	1211	1428

*Genomes of these six strains are all from this study.

Supplementary Table 2 Identification of key metabolites required for experimental validation via multiple analytical methods. ER, exchange reactions. Scenario 1: Each strain attains equal biomass growth; Scenario 2: Each strain within the community achieves growth (community-level optimal solution). For the exchange metabolites in multi-strain modeling, opposite patterns (OP) were observed: one was uptake (when C = 4), and the other was release (when C = 1). Using the SuperCC modeling framework, the exchange metabolites were simulated for six-strain combinations under two nutritional scenarios: (1) Minimal Medium (MM) supplemented with 100 mmol/gDW glucose (single carbon source, corresponding to C = 1); and (2) MM with 25 mmol/gDW each of glucose, citrate, acetate, and fumarate (quadruple carbon sources, corresponding to C = 4). Each metabolite was experimentally validated by supplementing it to the microbial consortium composed of strains A1, P1, C2, S4, B4, and L3 , which was inoculated into MM-10% P medium containing 10 ppm of TC and OTC. After 2 days of incubation, the residual concentrations of TC and OTC were measured, and the enhancement of degradation was calculated relative to the control without metabolite supplementation. TC, tetracycline; OTC, oxytetracycline. The data are presented as mean values (n = 4 biological independent replicates).

**Category**	**Compounds**	**Identified by metabolomics**	**Single-strain modeling**	**Multi-strain modeling**	**Enhanced degradation on TC**	**Enhanced degradation on OTC**
Amino Acids and Derivatives	L-Proline	√	√ (ER occur only in strains A1, P1, C2, and L3)	/	+3.98%	+4.34%
Amino Acids and Derivatives	N-Formyl-L-methionine	√	/	/	+1.50%	-0.24%
Amino Acids and Derivatives	L-Glutamate	/	√ (ER occur in all six strains)	√ (OP appear in Scenario 1; ER occur in Scenario 1 and 2)	+8.98%	+6.86%
Amino Acids and Derivatives	L-Phenylalanine	√	√ (ER occur only in strains A1, P1, C2, and S4)	√ (OP appear in Scenario 1; ER occur in Scenario 1 and 2)	+5.78%	+3.61%
Amino Acids and	Glycine	/	√	√	+8.98%	+8.27%

Derivatives			(ER occur only in strains A1, P1, C2, and L3)	(OP appear in Scenario 2; ER occur in Scenario 1 and 2)		
Amino Acids and Derivatives	L-Histidine	/	√ (ER occur only in strain A1)		+2.71%	+0.85%
Vitamins	Vitamin B3 (niacin)	√	√ (ER occur only in strain A1)		+21.36%	+6.43%
Vitamins	Vitamin B1	√	√ (ER occur only in strains A1, P1, C2, and L3)	√ (OP appear in Scenario 2; ER only occur in Scenario 2)	+18.91%	+7.70%
Vitamins	Vitamin D2	√	/	/	+15.66%	+8.69%
Organic Acids	Palmitate	√	√ (ER occur only in strains A1, P1, B4, and S4)	√ (OP appear in Scenario 2; ER only occur in Scenario 2)	+5.91%	+4.06%
Organic Acids	Myristic acid	/	√ (ER occur only in strains A1, P1, L3, B4, and S4)	√ (OP appear in Scenario 2; ER only occur in Scenario 2)	+12.85%	+8.02%
Organic Acids	Succinate	√	√ (ER occur only in strains A1, P1, C2, and S4)	/	+15.22%	+9.49%

Supplementary Table 3 Potentiator Contribution Index (PCI) for degradation rate and biomass under different pollutants.

Pollutants	PCI (%, degradation rate)	PCI (%, biomass)
DTAC	69.51886277	6.461001164
DBAC	79.39711011	
SM2	45.66666667	31.25
SMX	-23.24324324	
TC	394.9874687	37.88235294
OTC	96.28081906	
BO	10.78869859	32.18218218
QC	-0.463320463	

Supplementary Table 4 Experimental validation of sensitivity analysis of DHP-Com consortium for carbon source composition. Bacterial growth was monitored for 1800 min.

**Condition (Carbon sources)/mM**				**Mean Biomass**	**SD**	**Relative Change rate (%)**	**Robustness Coefficient (R)**
Glucose	Acetate	Citrate	Fumarate				
20	20	20	20	0.7301	0.0160	–	–
22	20	20	20	0.7094	0.0243	-2.84%	0.9716
18	20	20	20	0.7326	0.0224	+0.34%	0.9966
20	22	20	20	0.7280	0.0315	-0.29%	0.9971
20	18	20	20	0.7928	0.0335	+8.58%	0.9142
20	20	22	20	0.6668	0.0523	-8.67%	0.9133
20	20	18	20	0.6942	0.0424	-4.92%	0.9508
20	20	20	22	0.7752	0.0441	+6.17%	0.9383
20	20	20	18	0.7078	0.0172	-3.06%	0.9694
22	22	18	18	0.7170	0.0232	-1.80%	0.982
18	18	22	22	0.7266	0.0246	-0.48%	0.9952
22	18	18	22	0.7030	0.0196	-3.72%	0.9628
18	22	22	18	0.7312	0.0181	+0.15%	0.9985

Note: Relative change rate = (Biomass_perturbed - Biomass_baseline) / Biomass_baseline×100%

and defined the robustness coefficient as R = 1 - |Relative change rate| (where the absolute value accounts for both increases and decreases in biomass).

Supplementary Table 5 Modeling validation of sensitivity analysis of DHP-Com consortiumfor carbon source composition.

**Condition (Carbon sources)/mmol/g DW**	**Optimal Biomass**	**Relative Change rate (%)**	**Robustness Coefficient (R)**

Glucose	Citrate	Acetate	Fumarate			
25	25	25	25	421.812654823115	–	–
20	25	25	25	421.672057879753	-0.033331609%	0.999666684
30	25	25	25	421.953251766474	0.033331609%	0.999666684
25	20	25	25	421.812654823114	-2.42568E-13%	1
25	30	25	25	421.765789175328	-0.011110536%	0.999888895
25	25	20	25	421.765789175328	-0.011110536%	0.999888895
25	25	30	25	421.859520470903	0.011110536%	0.999888895
25	25	25	20	421.718923527538	-0.022221072%	0.999777789
25	25	25	30	421.906386118687	0.022221072%	0.999777789
20	20	30	30	421.812654823113	-4.7166E-13%	1
30	30	20	20	421.812654823071	-1.04304E-11%	1
20	30	20	30	421.718923527539	-0.022221072%	0.999777789
30	20	30	20	421.906386118687	0.022221072%	0.999777789
20	30	30	20	421.625192231966	-0.044442145%	0.999555579
30	20	20	30	422.000117414260	0.044442145%	0.999555579
20	20	20	20	421.531460936395	-0.066663217%	0.999333368
30	30	30	30	422.093848709832	0.066663217%	0.999333368

Note: Except for the change in the content of carbon source C, the contents of other inorganic substances remain unchanged. In particular, the content of NH_4^+ is always maintained at 100 mmol/g DW.
