# Supplementary material for: Potentiators empower synthetic microbiomes as silent guardians against co-contamination
Source: Nat Commun. 2025 Dec 31;17:1185. doi: 10.1038/s41467-025-67953-5 (PMC12858907; doi:10.1038/s41467-025-67953-5)
Supplement: Supplementary file 2 — Description of Additional Supplementary Files [file 41467_2025_67953_MOESM2_ESM.pdf]

Description Of Additional Supplementary Files

Supplementary Data 1: Top50 strains used in the study and general features of the metabolic models constructed for these strains.

Supplementary Data 2: Statistics of differential metabolite screening under different statistical criteria.

Supplementary Data 3: Biomass reaction composition for the reconstructed metabolic models. For each species, the upper section lists the ModelSEED compound identifiers for each component, while the lower section provides the corresponding chemical names.

Supplementary Data 4: Flux balance analysis illustrating interspecies metabolic exchange under the objective of equalizing biomass for each species in the community. Using the SuperCC modeling framework, the maximum biomass was simulated for six-strain combinations under two nutritional scenarios: (1) MM supplemented with 100 mmol/gDW glucose (single carbon source, C = 1); and (2) MM with 25 mmol/gDW each of glucose, citrate, acetate, and fumarate (quadruple carbon sources, C = 4). Substances with a light blue background represent metabolites unique to the C = 4 condition.

Supplementary Data 5:

Including:

File 1: Single strain models for six keystone strains and the corresponding six media.

File 2: Single strain models for the top 10% species.
