# Supplementary material for: Potentiators empower synthetic microbiomes as silent guardians against co-contamination
Source: Nat Commun. 2025 Dec 31;17:1185. doi: 10.1038/s41467-025-67953-5 (PMC12858907; doi:10.1038/s41467-025-67953-5)
Supplement: Supplementary file 3 — Supplementary Data 1 [file 41467_2025_67953_MOESM3_ESM.pdf]

Supplementary Data 1 Top50 strains used in the study and general features of the metabolic models constructed for these strains.

	Species	ASV	Genome accession*	Reactions	Exchange reactions	Transport reactions	Biochemical reactions	Metabolites	Biomass* (mmol/g DW)	Time (s)	Source
1	*Comamonas thiooxydans*	ASV1	GCA_000964545.1	1431	77	95	1259	1541	137.8226	0.008	TC, OTC, TCs
2	*Brevundimonas vancanneytii*	ASV2	GCA_003568665.1	1218	63	68	1087	1285	177.9518	0.008	TC, OTC, TCs
3	*Pseudomonas citronellolis*	ASV3	GCA_004745455.1	1666	115	135	1416	1697	213.9914	0.008	TC, OTC, TCs
4	*Elizabethkingia meningoseptica*	ASV4	GCA_900475375.1	1206	64	69	1073	1270	189.6203	0.008	TC, OTC, TCs
5	*Dysgonomonas mossii*	ASV5	GCA_000213575.1	1089	58	62	969	1202	79.8365	0.054	TC, OTC, TCs
6	*Stenotrophomonas nitritireducens*	ASV6	GCA_001431425.1	1297	72	79	1146	1394	179.283	0.008	TC, OTC, TCs
7	*Delftia tsuruhatensis*	ASV7	IMG_2675903019	1543	93	109	1341	1666	159.6958	0.006	TC, OTC, TCs
8	*Sphingobacterium daejeonense*	ASV8	GCA_002734235.1	1256	61	67	1128	1301	174.4804	0.008	TC, OTC, TCs
9	*Providencia stuartii*	ASV9	GCA_900455155.1	1259	100	109	1050	1321	178.8683	0.005	TC, OTC, TCs
10	*Pseudomonas citronellolis*	ASV10	GCA_004745455.1	1666	115	135	1416	1697	213.9914	0.008	TC, OTC, TCs
11	*Dysgonomonas gadei*	ASV11	GCA_000213555.1	1145	50	55	1040	1250	95.6267	0.008	TC, OTC, TCs

12	*Aquamicrobium lusatiense*	ASV12	GCA_029872115.1	1409	106	118	1185	1456	155.7246	0.009	TC, OTC, TCs
13	*Epilithonimonas pallida*	ASV13	IMG_2681813503	1212	65	68	1079	1268	165.2149	0.004	TC, OTC, TCs
14	*Brevundimonas terrae*	ASV14	GCA_011761985.1	1173	75	83	1015	1265	83.1846	0.008	TC, OTC, TCs
15	*Stenotrophomonas pictorum*	ASV15	GCA_001431585.1	1254	68	78	1108	1378	119.7931	0.008	TC, OTC, TCs
16	*Alcaligenes aquatilis*	ASV16	GCA_023373785.1	1389	96	107	1186	1511	138.0096	0.008	TC, OTC, TCs
17	*Achromobacter deleyi*	ASV17	GCA_000633435.1	1533	107	121	1305	1638	161.4146	0.008	TC, OTC, TCs
18	*Methylobacillus methanolivorans*	ASV18	GCA_044751345.1	1037	68	74	895	1165	50.4943	0.008	TC, OTC, TCs
19	*Taibaiella koreensis*	ASV19	GCA_003545715.1	1109	54	61	994	1198	90.6838	0.008	TC, OTC, TCs
20	*Achromobacter xylosoxidans*	ASV20	GCA_001457475.1	1531	108	120	1303	1631	163.1341	0.008	TC, OTC, TCs
21	*Leucobacter denitrificans*	ASV21	GCA_014396385.1	1180	87	92	1001	1247	73.3996	0.005	TC, OTC, TCs
22	*Klebsiella pasteurii*	ASV22	GCA_018139045.1	1771	130	148	1493	1714	356.5305	0.008	TC, OTC, TCs
23	*Bdellovibrio bacteriovorus*	ASV23	GCA_000196175.1	1112	47	52	1013	1230	53.9922	0.007	TC, OTC, TCs
24	*Stenotrophomonas nitritireducens*	ASV24	GCA_001431425.1	1297	72	79	1146	1394	179.283	0.008	TC, OTC, TCs
25	*Achromobacter ruhlandii*	ASV25	GCA_002082135.1	1493	92	105	1296	1606	167.253	0.012	TC, OTC, TCs

26	*Achromobacter insolitus*	ASV26	GCA_001971645.1	1513	90	103	1320	1640	171.8306	0.008	TC, OTC, TCs
27	*Pseudochrobactrum asaccharolyticum*	ASV27	GCA_003314995.1	1407	109	122	1176	1452	227.4055	0.008	TC, OTC, TCs
28	*Achromobacter animicus*	ASV28	GCA_902860125.1	1492	87	101	1304	1620	164.2228	0.024	TC, OTC, TCs
29	*Achromobacter xylosoxidans*	ASV29	GCA_001457475.1	1531	108	120	1303	1631	163.1341	0.008	TC, OTC, TCs
30	*Aquamicrobium defluvii*	ASV30	GCA_004363725.1	1394	89	102	1203	1474	197.758	0.013	TC, OTC, TCs
31	*Stenotrophomonas pictorum*	ASV31	GCA_001431585.1	1254	68	78	1108	1378	119.7931	0.008	OTC, TCs
32	*Elizabethkingia meningoseptica*	ASV32	GCA_900475375.1	1206	64	69	1073	1270	189.6203	0.008	TC, OTC
33	*Methylobacillus arboreus*	ASV33	GCA_020532725.1	1030	57	66	907	1164	74.7014	0.004	TC, OTC, TCs
34	*Sphingobacterium prati*	ASV34	GCA_013167215.1	1276	72	77	1127	1317	183.0856	0.008	TC, OTC, TCs
35	*Enterobacter asburiae*	ASV35	GCA_000952575.1	1598	115	132	1351	1579	335.1533	0.012	TC, OTC, TCs
36	*Achromobacter animicus*	ASV36	GCA_902860125.1	1492	87	101	1304	1620	164.2228	0.024	TC, OTC, TCs
37	*Elizabethkingia meningoseptica*	ASV37	GCA_900475375.1	1206	64	69	1073	1270	189.6203	0.008	TC, OTC
38	*Achromobacter anxifer*	ASV38	GCA_903652925.1	1548	106	119	1323	1656	166.5136	0.012	TC, OTC, TCs
39	*Alcaligenes aquatilis*	ASV39	GCA_023373785.1	1389	96	107	1186	1511	138.0096	0.008	OTC, TCs

40	*Bordetella hinzii*	ASV40	GCA_900637615.1	1450	86	96	1268	1579	163.8299	0.01	TC, OTC, TCs
41	*Chitinophaga alhagiae*	ASV41	GCA_003568665.1	1221	64	70	1087	1287	177.9518	0.008	TC, OTC, TCs
42	*Klebsiella aerogenes*	ASV42	GCA_000215745.1	1678	121	141	1416	1639	288.5965	0.008	TC, OTC, TCs
43	*Raoultella ornithinolytica*	ASV43	GCA_001598295.1	1765	133	156	1476	1699	333.9779	0.012	OTC, TCs
44	*Raoultella ornithinolytica*	ASV44	GCA_001598295.1	1765	133	156	1476	1699	333.9779	0.012	TC, OTC, TCs
45	*Bosea robiniae*	ASV45	IMG_2675903146	1445	86	99	1260	1510	174.8173	0.024	TC, OTC, TCs
46	*Bordetella petrii*	ASV46	GCA_000067205.1	1362	84	93	1185	1470	158.9216	0.011	TC, OTC, TCs
47	*Leucobacter komagatae*	ASV47	GCA_006716085.1	1244	86	101	1057	1296	107.9772	0.004	OTC
48	*Bordetella petrii*	ASV48	GCA_000067205.1	1362	84	93	1185	1470	158.9216	0.011	OTC
49	*Raoultella ornithinolytica*	ASV49	GCA_001598295.1	1765	133	156	1476	1699	333.9779	0.012	OTC
50	*Leucobacter chromiireducens*	ASV50	GCA_016758195.1	1227	90	100	1037	1295	106.8899	0.008	OTC
51	*Bordetella trematum*	ASV51	GCA_900445945.1	1364	80	91	1193	1489	123.0272	0.008	TC, OTC, TCs
52	*Mycobacterium mageritense*	ASV52	GCA_010727475.1	1621	106	125	1390	1603	273.4657	0.012	TC, OTC
53	*Phytobacter ursingii*	ASV53	GCA_001022135.1	1680	112	128	1440	1670	303.1863	0.012	OTC, TCs

54	*Devosia equisanguinis*	ASV54	GCA_900631955.1	1320	86	97	1137	1388	117.4912	0.008	TC, OTC, TCs
55	*Methylobacillus methanolivorans*	ASV55	GCA_044751345.1	1037	68	74	895	1165	50.4943	0.008	TC, TCs
56	*Sheuella amnicola*	ASV56	GCA_010499255.1	1240	66	78	1096	1330	88.3359	0.009	TC, OTC, TCs
57	*Chitinophaga dinghuensis*	ASV57	GCA_003259435.1	1310	63	67	1180	1373	219.3401	0.012	TC, OTC, TCs
58	*Elizabethkingia anophelis subsp anophelis*	ASV58	GCA_002023665.2	1246	83	88	1075	1288	211.0822	0.008	OTC
59	*Chitinophaga niastensis*	ASV59	GCA_003014755.1	1303	65	72	1166	1389	191.3442	0.008	TC, OTC
60	*Pigmentiphaga humi*	ASV62	GCA_900606115.1	1398	84	92	1222	1535	132.9528	0.008	TC, OTC, TCs
61	*Pseudomonas paraeruginosa*	ASV68	GCA_900706985.1	1520	86	98	1336	1578	200.7092	0.008	TC
62	*Alcaligenes faecalis subsp faecalis*	ASV72	GCA_002443155.1	1365	96	107	1162	1472	138.0096	0.032	TCs
63	*Methylobacillus methanolivorans*	ASV75	GCA_044751345.1	1037	68	74	895	1165	50.4943	0.008	TC, TCs
64	*Stenotrophomonas pictorum*	ASV80	GCA_001431585.1	1254	68	78	1108	1378	119.7931	0.008	TCs
65	*Shinella granuli*	ASV82	GCA_004341885.1	1578	96	108	1374	1654	208.0796	0.012	TC, OTC, TCs
66	*Elizabethkingia meningoseptica*	ASV83	GCA_900475375.1	1206	64	69	1073	1270	189.6203	0.008	TC

67	*Dielma fastidiosa*	ASV86	GCA_000313565.2	986	82	82	822	1036	17.5905	0.008	TC, TCs
68	*Elizabethkingia meningoseptica*	ASV90	GCA_900475375.1	1206	64	69	1073	1270	189.6203	0.008	TC
69	*Pseudomonas schmalbachii*	ASV106	GCA_017589465.1	1481	90	105	1286	1539	200.1956	0.004	TC
70	*Raoultella ornithinolytica*	ASV108	GCA_001598295.1	1765	133	156	1476	1699	333.9779	0.012	TC

*Genomes of the mostly closely related species of each strain with available genome sequences were chosen for model construction.

*Biomass in complete medium.
