# Supplementary material for: Potentiators empower synthetic microbiomes as silent guardians against co-contamination
Source: Nat Commun. 2025 Dec 31;17:1185. doi: 10.1038/s41467-025-67953-5 (PMC12858907; doi:10.1038/s41467-025-67953-5)
Supplement: Supplementary file 4 — Supplementary Data 2 [file 41467_2025_67953_MOESM4_ESM.pdf]

Supplementary Data 2 Statistics of differential metabolite screening under different statistical criteria (Student's t-test, two-sided).

**Group**	**Screening criteria for differential metabolites**					
	p-value < 0.05, VIP score ≥ 1, and fold change ≥ 1			FRD < 0.05, VIP score ≥ 1, and fold change ≥ 1		
	Number of up-regulated metabolites	Number of down-regulated metabolites	All	Number of up-regulated metabolites	Number of down-regulated metabolites	All
L_TCs vs L_CK	559	316	875	552	306	858
B_TCs vs B_CK	510	177	687	506	177	683
S_TCs vs S_CK	507	163	670	506	159	665
P_TCs vs P_CK	498	165	663	495	164	659
A_TCs vs A_CK	543	154	697	537	147	684
C_TCs vs C_CK	506	130	636	503	128	631
AP_TCs vs AP_CK	586	191	777	584	187	771
PB_TCs vs PB_CK	582	197	779	579	193	772
AS_TCs vs AS_CK	598	152	750	593	148	741
PC_TCs vs PC_CK	545	157	702	540	147	687
PS_TCs vs PS_CK	535	190	725	533	186	719
CB_TCs vs CB_CK	597	172	769	588	168	756
AB_TCs vs AB_CK	617	109	726	615	107	722
SB_TCs vs SB_CK	522	169	691	522	168	690

CS_TCs vs CS_CK	539	145	684	534	143	677
AC_TCs vs AC_CK	583	129	712	576	123	699
CSB_TCs vs CSB_CK	555	219	774	548	193	741
PCB_TCs vs PCB_CK	608	168	776	600	163	763
ACS_TCs vs ACS_CK	622	141	763	612	136	748
PSB_TCs vs PSB_CK	566	164	730	564	161	725
APC_TCs vs APC_CK	762	239	1001	759	237	996
APB_TCs vs APB_CK	619	198	817	615	192	807
ACB_TCs vs ACB_CK	633	134	767	632	134	766
APS_TCs vs APS_CK	598	159	757	589	157	746
PCS_TCs vs PCS_CK	542	169	711	540	162	702
ASB_TCs vs ASB_CK	603	104	707	573	86	659

Note: TCs: Treatment group exposed to a combination of tetracycline and oxytetracycline. CK: Control group without pollutant exposure. The letters (e.g., L, B, S) denote different bacterial consortia: L3, B4, S4, A1, C2, P1.
