# Supplementary material for: Potentiators empower synthetic microbiomes as silent guardians against co-contamination
Source: Nat Commun. 2025 Dec 31;17:1185. doi: 10.1038/s41467-025-67953-5 (PMC12858907; doi:10.1038/s41467-025-67953-5)
Supplement: Supplementary file 5 — Supplementary Data 3 [file 41467_2025_67953_MOESM5_ESM.pdf]

Supplementary Data 3 Biomass reaction composition for the reconstructed metabolic models. For each species, the upper section lists the ModelSEED compound identifiers for each component, while the lower section provides the corresponding chemical names.

Strains	Biomass reactions
A1	40.1102 cpd00002[c] + 0.50987 cpd00033[c] + 0.00309647 cpd00042[c] + 0.00309647 cpd00017[c] + 0.127801 cpd00060[c] + 0.179456 cpd00054[c] + 0.00309647 cpd00010[c] + 35.5403 cpd00001[c] + 0.219088 cpd00023[c] + 0.0761465 cpd00084[c] + 0.00309647 cpd00003[c] + 0.0160207 cpd00356[c] + 0.00309647 cpd00056[c] + 0.00309647 cpd00006[c] + 0.285438 cpd00039[c] + 0.00309647 cpd11493[c] + cpd17042[c] + 0.00309647 cpd15560[c] + 0.200831 cpd00041[c] + 0.135407 cpd00038[c] + 0.00309647 cpd00048[c] + 0.0841036 cpd00052[c] + 0.00309647 cpd00201[c] + 0.00309647 cpd00099[c] + 0.00309647 cpd00015[c] + 0.00309647 cpd00118[c] + 0.00309647 cpd00345[c] + 0.154519 cpd00066[c] + 0.427934 cpd00035[c] + 0.00309647 cpd00016[c] + 0.0792636 cpd00119[c] + 0.219088 cpd00053[c] + 0.200831 cpd00132[c] + 0.00309647 cpd15500[c] + 0.00309647 cpd15352[c] + 0.00309647 cpd00264[c] + 0.010648 cpd15533[c] + 0.00309647 cpd00063[c] + 0.00309647 cpd00058[c] + 0.00309647 cpd00087[c] + 0.246697 cpd00051[c] + 0.00309647 cpd00220[c] + 0.375389 cpd00107[c] + 0.0160207 cpd00357[c] + 0.184355 cpd00129[c] + 0.0160207 cpd00241[c] + 0.120677 cpd00069[c] + 0.241799 cpd00322[c] + 0.00309647 cpd00034[c] + 0.0160207 cpd00115[c] + 0.211073 cpd00161[c] + 0.0908319 cpd00062[c] + cpd17043[c] + 0.0472019 cpd00065[c] + 0.352233 cpd00156[c] + cpd17041[c] + 0.010648 cpd15540[c] + 0.010648 cpd15722[c] + 0.00309647 cpd10516[c] + 0.00309647 cpd00254[c] + 0.00309647 cpd00205[c] + 0.010648 cpd15723[c] + 0.00309647 cpd00149[c] + 0.010648 cpd15696[c] + 0.00309647 cpd00557[c] + 0.00309647 cpd10515[c] + 0.0250106 cpd02229[c] + 0.0250106 cpd15665[c] + 0.010648 cpd15695[c] + 0.00309647 cpd00028[c] + 0.010648 cpd15794[c] + 0.0250106 cpd15432[c] + 0.00309647 cpd00030[c] + 0.010648 cpd15793[c] + 0.00309647 cpd00166[c] + 0.010648 cpd15795[c] -> 0.484601 cpd00012[c] + 40 cpd00067[c] + 40 cpd00008[c] + 39.9969 cpd00009[c] + 0.0250106 cpd15666[c] + 0.00309647 cpd12370[c] + cpd11416[c] + 0.00309647 cpd01997[c] + 0.00309647 cpd03422[c]
	40.1102 ATP[c] + 0.50987 Glycine[c] + 0.00309647 Glutathione[c] + 0.00309647 Acylcarnitine[c] + 0.127801 L-Methionine[c] + 0.179456 L-Serine[c] + 0.00309647 Coenzyme A[c] + 35.5403 H2O[c] + 0.219088 Glutamate[c] + 0.0761465 L-Cysteine[c] + 0.00309647 NAD[c] + 0.0160207 Deoxycytidine 5'-triphosphate[c] + 0.00309647 Thiamine diphosphate[c] + 0.00309647 NADP[c] + 0.285438 L-Lysine[c] + 0.00309647 ACP[c] + DNA replication[c] + 0.00309647 Ubiquinone-8[c] + 0.200831 L-Aspartate[c] + 0.135407 GTP[c] + 0.00309647 Sulfate[c] + 0.0841036 CTP[c] + 0.00309647 10-formyl-THF mono-L-glutamate[c] + 0.00309647 Chloride ion[c] + 0.00309647 Flavin adenine dinucleotide[c] + 0.00309647 Putrescine[c] + 0.00309647 5-Methyltetrahydrofolate[c] + 0.154519 L-Phenylalanine[c] + 0.427934 L-Alanine[c] + 0.00309647 Pyridoxal phosphate[c] + 0.0792636 L-Histidine[c] + 0.219088 L-Glutamine[c] + 0.200831 L-Asparagine[c] + 0.00309647 Menaquinone 8[c] + 0.00309647 2-Demethylmenaquinone 8[c] + 0.00309647 Spermidine[c] + 0.010648 Phosphatidylethanolamine dioctadecanoyl[c] + 0.00309647 Ca(2+)[c] + 0.00309647 Cu(II)[c] + 0.00309647 Tetrahydrofolate[c] + 0.246697 L-Arginine[c] + 0.00309647 Riboflavin[c] + 0.375389 L-Leucine[c] + 0.0160207

	Deoxythymidine triphosphate[c] + 0.184355 L-Proline[c] + 0.0160207 dGTP[c] + 0.120677 L-Tyrosine[c] + 0.241799 L-Isoleucine[c] + 0.00309647 Zn(II)[c] + 0.0160207 dATP[c] + 0.211073 L-Threonine [c] + 0.0908319 UTP [c] + RNA transcription[c] + 0.0472019 L-Tryptophan [c] + 0.352233 L-Valine [c] + Protein biosynthesis[c] + 0.010648 Phosphatidylglycerol dioctadecanoyl[c] + 0.010648 Diisoheptadecanoylphosphatidylglycerol[c] + 0.00309647 Fe(III) [c] + 0.00309647 Mg(2+)[c] + 0.00309647 K(+)[c] + 0.010648 Dianteisoheptadecanoylphosphatidylglycerol[c] + 0.00309647 Co(II)[c] + 0.010648 Dianteisoheptadecanoylphosphatidylethanolamine[c] + 0.00309647 Siroheme[c] + 0.00309647 Fe(II)[c] + 0.0250106 Calomide[c] + 0.0250106 Peptidoglycan polymer (n subunits)[c] + 0.010648 Diisoheptadecanoylphosphatidylethanolamine[c] + 0.00309647 Heme[c] + 0.010648 Isoheptadecanoylcardiolipin[c] + 0.0250106 Core oligosaccharide lipid A[c] + 0.00309647 Mn(II)[c] + 0.010648 Stearoylcardiolipin[c] + 0.00309647 Calomide[c] + 0.010648 Anteisoheptadecanoylcardiolipin[c] -> 0.484601 Diphosphoric acid[c] + 40 H(+)[c] + 40 ADP[c] + 39.9969 Phosphate[c] + 0.0250106 Peptidoglycan polymer (n-1 subunits)[c] + 0.00309647 apo-ACP[c] + Biomass[c] + 0.00309647 Dimethylbenzimidazole[c] + 0.00309647 Cobinamide[c]
**P1**	40.1102 cpd00002[c] + 0.50987 cpd00033[c] + 0.00309647 cpd00042[c] + 35.5403 cpd00001[c] + 0.200831 cpd00041[c] + 0.00309647 cpd00017[c] + 0.127801 cpd00060[c] + 0.179456 cpd00054[c] + 0.00309647 cpd00010[c] + 0.219088 cpd00023[c] + 0.0761465 cpd00084[c] + 0.00309647 cpd00003[c] + 0.00309647 cpd00015[c] + 0.0160207 cpd00356[c] + 0.00309647 cpd00056[c] + 0.00309647 cpd00006[c] + 0.00309647 cpd11493[c] + cpd17042[c] + 0.00309647 cpd15560[c] + 0.135407 cpd00038[c] + 0.00309647 cpd00048[c] + 0.0841036 cpd00052[c] + 0.00309647 cpd00201[c] + 0.00309647 cpd00118[c] + 0.00309647 cpd00264[c] + 0.00309647 cpd00345[c] + 0.154519 cpd00066[c] + 0.184355 cpd00129[c] + 0.00309647 cpd00016[c] + 0.0792636 cpd00119[c] + 0.427934 cpd00035[c] + 0.00309647 cpd15500[c] + 0.00309647 cpd15352[c] + 0.010648 cpd15533[c] + 0.00309647 cpd00058[c] + 0.00309647 cpd00087[c] + 0.246697 cpd00051[c] + 0.00309647 cpd00220[c] + 0.200831 cpd00132[c] + 0.0160207 cpd00357[c] + 0.219088 cpd00053[c] + 0.0160207 cpd00241[c] + 0.120677 cpd00069[c] + 0.241799 cpd00322[c] + 0.00309647 cpd00034[c] + 0.285438 cpd00039[c] + 0.0160207 cpd00115[c] + 0.211073 cpd00161[c] + 0.00309647 cpd10516[c] + 0.0908319 cpd00062[c] + cpd17043[c] + 0.0472019 cpd00065[c] + 0.352233 cpd00156[c] + 0.375389 cpd00107[c] + cpd17041[c] + 0.010648 cpd15540[c] + 0.010648 cpd15722[c] + 0.00309647 cpd00254[c] + 0.00309647 cpd00205[c] + 0.00309647 cpd00099[c] + 0.00309647 cpd00149[c] + 0.010648 cpd15723[c] + 0.010648 cpd15696[c] + 0.0250106 cpd15432[c] + 0.00309647 cpd00030[c] + 0.0250106 cpd02229[c] + 0.0250106 cpd15665[c] + 0.010648 cpd15695[c] + 0.00309647 cpd10515[c] + 0.00309647 cpd00028[c] + 0.00309647 cpd00063[c] + 0.010648 cpd15793[c] + 0.010648 cpd15795[c] + 0.00309647 cpd00166[c] + 0.010648 cpd15794[c] + 0.00309647 cpd00557[c] -> 0.484601 cpd00012[c] + 40 cpd00067[c] + 40 cpd00008[c] + 39.9969 cpd00009[c] + 0.00309647 cpd12370[c] + 0.00309647 cpd03422[c] + 0.0250106 cpd15666[c] + 0.00309647 cpd01997[c] + cpd11416[c]

	40.1102 ATP[c] + 0.50987 Glycine[c] + 0.00309647 Glutathione[c] + 35.5403 H2O[c] + 0.200831 L-Aspartate[c] + 0.00309647 Acylcarnitine[c] + 0.127801 L-Methionine[c] + 0.179456 L-Serine[c] + 0.00309647 Coenzyme A[c] + 0.219088 Glutamate[c] + 0.0761465 L-Cysteine[c] + 0.00309647 NAD[c] + 0.00309647 Flavin adenine dinucleotide[c] + 0.0160207 Deoxycytidine 5'-triphosphate[c] + 0.00309647 Thiamine diphosphate[c] + 0.00309647 NADP[c] + 0.00309647 ACP[c] + DNA replication[c] + 0.00309647 Ubiquinone-8[c] + 0.135407 GTP[c] + 0.00309647 Sulfate[c] + 0.0841036 CTP[c] + 0.00309647 10-formyl-THF mono-L-glutamate[c] + 0.00309647 Putrescine[c] + 0.00309647 Spermidine[c] + 0.00309647 5-Methyltetrahydrofolate[c] + 0.154519 L-Phenylalanine[c] + 0.184355 L-Proline[c] + 0.00309647 Pyridoxal phosphate[c] + 0.0792636 L-Histidine[c] + 0.427934 L-Alanine[c] + 0.00309647 Menaquinone 8[c] + 0.00309647 2-Demethylmenaquinone 8[c] + 0.010648 Phosphatidylethanolamine dioctadecanoyl[c] + 0.00309647 Cu(II)[c] + 0.00309647 Tetrahydrofolate[c] + 0.246697 L-Arginine[c] + 0.00309647 Riboflavin[c] + 0.200831 L-Asparagine[c] + 0.0160207 Deoxythymidine triphosphate[c] + 0.219088 L-Glutamine[c] + 0.0160207 dGTP[c] + 0.120677 L-Tyrosine[c] + 0.241799 L-Isoleucine[c] + 0.00309647 Zn(II)[c] + 0.285438 L-Lysine[c] + 0.0160207 dATP[c] + 0.211073 L-Threonine [c] + 0.00309647 Fe(III) [c] + 0.0908319 UTP [c] + RNA transcription[c] + 0.0472019 L-Tryptophan [c] + 0.352233 L-Valine [c] + 0.375389 L-Leucine[c] + Protein biosynthesis[c] + 0.010648 Phosphatidylglycerol dioctadecanoyl[c] + 0.010648 Diisoheptadecanoylphosphatidylglycerol[c] + 0.00309647 Mg(2+)[c] + 0.00309647 K(+)[c] + 0.00309647 Chloride ion[c] + 0.00309647 Co(II)[c] + 0.010648 Dianteisoheptadecanoylphosphatidylglycerol[c] + 0.010648 Dianteisoheptadecanoylphosphatidylethanolamine[c] + 0.0250106 Core oligosaccharide lipid A[c] + 0.00309647 Mn(II)[c] + 0.0250106 Calomide[c] + 0.0250106 Peptidoglycan polymer (n subunits)[c] + 0.010648 Diisoheptadecanoylphosphatidylethanolamine[c] + 0.00309647 Fe(II)[c] + 0.00309647 Heme[c] + 0.00309647 Ca(2+)[c] + 0.010648 Stearoylcardiolipin[c] + 0.010648 Anteisoheptadecanoylcardiolipin[c] + 0.00309647 Calomide[c] + 0.010648 Isoheptadecanoylcardiolipin[c] + 0.00309647 Siroheme[c] -> 0.484601 Diphosphoric acid[c] + 40 H(+)[c] + 40 ADP[c] + 39.9969 Phosphate[c] + 0.00309647 apo-ACP[c] + 0.00309647 Cobinamide[c] + 0.0250106 Peptidoglycan polymer (n-1 subunits)[c] + 0.00309647 Dimethylbenzimidazole[c] + Biomass[c]
**C2**	40.1102 cpd00002[c] + 0.50987 cpd00033[c] + 0.00309647 cpd00042[c] + 35.5403 cpd00001[c] + 0.200831 cpd00041[c] + 0.00309647 cpd00017[c] + 0.127801 cpd00060[c] + 0.179456 cpd00054[c] + 0.00309647 cpd00010[c] + 0.219088 cpd00023[c] + 0.0761465 cpd00084[c] + 0.00309647 cpd00003[c] + 0.00309647 cpd00015[c] + 0.0160207 cpd00356[c] + 0.00309647 cpd00056[c] + 0.00309647 cpd00006[c] + 0.285438 cpd00039[c] + 0.00309647 cpd11493[c] + cpd17042[c] + 0.00309647 cpd15560[c] + 0.135407 cpd00038[c] + 0.00309647 cpd00048[c] + 0.0841036 cpd00052[c] + 0.00309647 cpd00201[c] + 0.00309647 cpd00099[c] + 0.00309647 cpd00118[c] + 0.00309647 cpd00345[c] + 0.154519 cpd00066[c] + 0.184355 cpd00129[c] + 0.427934 cpd00035[c] + 0.00309647 cpd00016[c] + 0.0792636 cpd00119[c] + 0.00309647 cpd15500[c] + 0.00309647 cpd15352[c] + 0.010648 cpd15533[c] + 0.00309647 cpd00058[c] + 0.00309647 cpd00087[c] + 0.246697 cpd00051[c] + 0.00309647 cpd00220[c] + 0.0160207 cpd00357[c] + 0.219088 cpd00053[c] + 0.120677 cpd00069[c] + 0.241799 cpd00322[c] + 0.0160207 cpd00115[c] + 0.211073 cpd00161[c] + 0.0908319 cpd00062[c] + cpd17043[c] + 0.0472019 cpd00065[c] + 0.352233 cpd00156[c] + 0.375389 cpd00107[c] + cpd17041[c] + 0.010648

	cpd15540[c] + 0.010648 cpd15722[c] + 0.0160207 cpd00241[c] + 0.00309647 cpd00254[c] + 0.00309647 cpd00205[c] + 0.00309647 cpd00149[c] + 0.010648 cpd15723[c] + 0.00309647 cpd00166[c] + 0.00309647 cpd10516[c] + 0.010648 cpd15696[c] + 0.00309647 cpd00264[c] + 0.0250106 cpd02229[c] + 0.0250106 cpd15665[c] + 0.200831 cpd00132[c] + 0.010648 cpd15695[c] + 0.00309647 cpd10515[c] + 0.00309647 cpd00028[c] + 0.010648 cpd15793[c] + 0.00309647 cpd00030[c] + 0.00309647 cpd00063[c] + 0.00309647 cpd00557[c] + 0.0250106 cpd15432[c] + 0.010648 cpd15795[c] + 0.00309647 cpd00034[c] + 0.010648 cpd15794[c] -> 0.484601 cpd00012[c] + 40 cpd00067[c] + 40 cpd00008[c] + 39.9969 cpd00009[c] + 0.00309647 cpd03422[c] + 0.00309647 cpd01997[c] + 0.0250106 cpd15666[c] + cpd11416[c] + 0.00309647 cpd12370[c]
	40.1102 ATP[c] + 0.50987 Glycine[c] + 0.00309647 Glutathione[c] + 35.5403 H2O[c] + 0.200831 L-Aspartate[c] + 0.00309647 Acylcarnitine[c] + 0.127801 L-Methionine[c] + 0.179456 L-Serine[c] + 0.00309647 Coenzyme A[c] + 0.219088 Glutamate[c] + 0.0761465 L-Cysteine[c] + 0.00309647 NAD[c] + 0.00309647 Flavin adenine dinucleotide[c] + 0.0160207 Deoxycytidine 5'-triphosphate[c] + 0.00309647 Thiamine diphosphate[c] + 0.00309647 NADP[c] + 0.285438 L-Lysine[c] + 0.00309647 ACP[c] + DNA replication[c] + 0.00309647 Ubiquinone-8[c] + 0.135407 GTP[c] + 0.00309647 Sulfate[c] + 0.0841036 CTP[c] + 0.00309647 10-formyl-THF mono-L-glutamate[c] + 0.00309647 Chloride ion[c] + 0.00309647 Putrescine[c] + 0.00309647 5-Methyltetrahydrofolate[c] + 0.154519 L-Phenylalanine[c] + 0.184355 L-Proline[c] + 0.427934 L-Alanine[c] + 0.00309647 Pyridoxal phosphate[c] + 0.0792636 L-Histidine[c] + 0.00309647 Menaquinone 8[c] + 0.00309647 2-Demethylmenaquinone 8[c] + 0.010648 Phosphatidylethanolamine dioctadecanoyl[c] + 0.00309647 Cu(II)[c] + 0.00309647 Tetrahydrofolate[c] + 0.246697 L-Arginine[c] + 0.00309647 Riboflavin[c] + 0.0160207 Deoxythymidine triphosphate[c] + 0.219088 L-Glutamine[c] + 0.120677 L-Tyrosine[c] + 0.241799 L-Isoleucine[c] + 0.0160207 dATP[c] + 0.211073 L-Threonine [c] + 0.0908319 UTP [c] + RNA transcription[c] + 0.0472019 L-Tryptophan [c] + 0.352233 L-Valine [c] + 0.375389 L-Leucine[c] + Protein biosynthesis[c] + 0.010648 Phosphatidylglycerol dioctadecanoyl[c] + 0.010648 Diisoheptadecanoylphosphatidylglycerol[c] + 0.0160207 dGTP[c] + 0.00309647 Mg(2+)[c] + 0.00309647 K(+)[c] + 0.00309647 Co(II)[c] + 0.010648 Dianteisoheptadecanoylphosphatidylglycerol[c] + 0.00309647 Calomide[c] + 0.00309647 Fe(III) [c] + 0.010648 Dianteisoheptadecanoylphosphatidylethanolamine[c] + 0.00309647 Spermidine[c] + 0.0250106 Calomide[c] + 0.0250106 Peptidoglycan polymer (n subunits)[c] + 0.200831 L-Asparagine[c] + 0.010648 Diisoheptadecanoylphosphatidylethanolamine[c] + 0.00309647 Fe(II)[c] + 0.00309647 Heme[c] + 0.010648 Stearoylcardiolipin[c] + 0.00309647 Mn(II)[c] + 0.00309647 Ca(2+)[c] + 0.00309647 Siroheme[c] + 0.0250106 Core oligosaccharide lipid A[c] + 0.010648 Anteisoheptadecanoylcardiolipin[c] + 0.00309647 Zn(II)[c] + 0.010648 Isoheptadecanoylcardiolipin[c] -> 0.484601 Diphosphoric acid[c] + 40 H(+)[c] + 40 ADP[c] + 39.9969 Phosphate[c] + 0.00309647 Cobinamide[c] + 0.00309647 Dimethylbenzimidazole[c] + 0.0250106 Peptidoglycan polymer (n-1 subunits)[c] + Biomass[c] + 0.00309647 apo-ACP[c]

L3	0.00309647 cpd00056[c] + 35.5403 cpd00001[c] + 0.200831 cpd00041[c] + 0.179456 cpd00054[c] + 0.00309647 cpd00010[c] + 40.1102 cpd00002[c] + 0.00309647 cpd00015[c] + 0.0160207 cpd00356[c] + 0.00309647 cpd00006[c] + 0.00309647 cpd00003[c] + 0.285438 cpd00039[c] + 0.00309647 cpd11493[c] + cpd17042[c] + 0.00309647 cpd15560[c] + 0.50987 cpd00033[c] + 0.0841036 cpd00052[c] + 0.0761465 cpd00084[c] + 0.219088 cpd00023[c] + 0.00309647 cpd00201[c] + 0.00309647 cpd00017[c] + 0.00309647 cpd00118[c] + 0.00309647 cpd00345[c] + 0.154519 cpd00066[c] + 0.00309647 cpd00016[c] + 0.120677 cpd00069[c] + 0.135407 cpd00038[c] + 0.427934 cpd00035[c] + 0.00309647 cpd15500[c] + 0.00309647 cpd15352[c] + 0.00309647 cpd00058[c] + 0.00309647 cpd00087[c] + 0.246697 cpd00051[c] + 0.00309647 cpd00220[c] + 0.375389 cpd00107[c] + 0.0160207 cpd00357[c] + 0.219088 cpd00053[c] + 0.184355 cpd00129[c] + 0.127801 cpd00060[c] + 0.241799 cpd00322[c] + 0.00309647 cpd00034[c] + 0.0160207 cpd00115[c] + 0.211073 cpd00161[c] + 0.00309647 cpd10516[c] + 0.0908319 cpd00062[c] + cpd17043[c] + 0.00309647 cpd00042[c] + 0.00309647 cpd00264[c] + 0.352233 cpd00156[c] + 0.0160207 cpd00241[c] + cpd17041[c] + 0.0472019 cpd00065[c] + 0.0792636 cpd00119[c] + 0.00309647 cpd00254[c] + 0.00309647 cpd00205[c] + 0.00309647 cpd00030[c] + 0.00309647 cpd00048[c] + 0.0250106 cpd02229[c] + 0.0250106 cpd15665[c] + 0.200831 cpd00132[c] + 0.010648 cpd15533[c] + 0.00309647 cpd00028[c] + 0.00309647 cpd00149[c] + 0.00309647 cpd00063[c] + 0.010648 cpd15795[c] + 0.00309647 cpd10515[c] + 0.010648 cpd15696[c] + 0.00309647 cpd00166[c] + 0.010648 cpd15793[c] + 0.010648 cpd15794[c] + 0.00309647 cpd00099[c] + 0.0250106 cpd15432[c] + 0.010648 cpd15540[c] + 0.00309647 cpd00557[c] + 0.010648 cpd15723[c] + 0.010648 cpd15722[c] + 0.010648 cpd15695[c] -> 0.484601 cpd00012[c] + 40 cpd00067[c] + 40 cpd00008[c] + 39.9969 cpd00009[c] + 0.0250106 cpd15666[c] + 0.00309647 cpd03422[c] + cpd11416[c] + 0.00309647 cpd01997[c] + 0.00309647 cpd12370[c]
	0.00309647 Thiamine diphosphate[c] + 35.5403 H2O[c] + 0.200831 L-Aspartate[c] + 0.179456 L-Serine[c] + 0.00309647 Coenzyme A[c] + 40.1102 ATP[c] + 0.00309647 Flavin adenine dinucleotide[c] + 0.0160207 Deoxycytidine 5'-triphosphate[c] + 0.00309647 NADP[c] + 0.00309647 NAD[c] + 0.285438 L-Lysine[c] + 0.00309647 ACP[c] + DNA replication[c] + 0.00309647 Ubiquinone-8[c] + 0.50987 Glycine[c] + 0.0841036 CTP[c] + 0.0761465 L-Cysteine[c] + 0.219088 Glutamate[c] + 0.00309647 10-formyl-THF mono-L-glutamate[c] + 0.00309647 Acylcarnitine[c] + 0.00309647 Putrescine[c] + 0.00309647 5-Methyltetrahydrofolate[c] + 0.154519 L-Phenylalanine[c] + 0.00309647 Pyridoxal phosphate[c] + 0.120677 L-Tyrosine[c] + 0.135407 GTP[c] + 0.427934 L-Alanine[c] + 0.00309647 Menaquinone 8[c] + 0.00309647 2-Demethylmenaquinone 8[c] + 0.00309647 Cu(II)[c] + 0.00309647 Tetrahydrofolate[c] + 0.246697 L-Arginine[c] + 0.00309647 Riboflavin[c] + 0.375389 L-Leucine[c] + 0.0160207 Deoxythymidine triphosphate[c] + 0.219088 L-Glutamine[c] + 0.184355 L-Proline[c] + 0.127801 L-Methionine[c] + 0.241799 L-Isoleucine[c] + 0.00309647 Zn(II)[c] + 0.0160207 dATP[c] + 0.211073 L-Threonine [c] + 0.00309647 Fe(III) [c] + 0.0908319 UTP [c] + RNA transcription[c] + 0.00309647 Glutathione[c] + 0.00309647 Spermidine[c] + 0.352233 L-Valine [c] + 0.0160207 dGTP[c] + Protein biosynthesis[c] + 0.0472019 L-Tryptophan [c] + 0.0792636 L-Histidine[c] + 0.00309647 Mg(2+)[c] + 0.00309647 K(+)[c] + 0.00309647 Mn(II)[c] + 0.00309647 Sulfate[c] + 0.0250106 Calomide[c] + 0.0250106 Peptidoglycan polymer (n subunits)[c] + 0.200831 L-Asparagine[c] + 0.010648 Phosphatidylethanolamine

	dioctadecanoyl[c] + 0.00309647 Heme[c] + 0.00309647 Co(II)[c] + 0.00309647 Ca(2+)[c] + 0.010648 Anteisoheptadecanoylcardiolipin[c] + 0.00309647 Fe(II)[c] + 0.010648 Dianteisoheptadecanoylphosphatidylethanolamine[c] + 0.00309647 Calomide[c] + 0.010648 Stearoylcardiolipin[c] + 0.010648 Isoheptadecanoylcardiolipin[c] + 0.00309647 Chloride ion[c] + 0.0250106 Core oligosaccharide lipid A[c] + 0.010648 Phosphatidylglycerol dioctadecanoyl[c] + 0.00309647 Siroheme[c] + 0.010648 Dianteisoheptadecanoylphosphatidylglycerol[c] + 0.010648 Diisoheptadecanoylphosphatidylglycerol[c] + 0.010648 Diisoheptadecanoylphosphatidylethanolamine[c] -> 0.484601 Diphosphoric acid[c] + 40 H(+)[c] + 40 ADP[c] + 39.9969 Phosphate[c] + 0.0250106 Peptidoglycan polymer (n-1 subunits)[c] + 0.00309647 Cobinamide[c] + Biomass[c] + 0.00309647 Dimethylbenzimidazole[c] + 0.00309647 apo-ACP[c]
**B4**	40.1102 cpd00002[c] + 0.50987 cpd00033[c] + 0.00309647 cpd00042[c] + 0.00309647 cpd00056[c] + 35.5403 cpd00001[c] + 0.200831 cpd00041[c] + 0.00309647 cpd00017[c] + 0.127801 cpd00060[c] + 0.179456 cpd00054[c] + 0.00309647 cpd00010[c] + 0.219088 cpd00023[c] + 0.0761465 cpd00084[c] + 0.00309647 cpd00003[c] + 0.0160207 cpd00356[c] + 0.00309647 cpd00006[c] + 0.00309647 cpd11493[c] + cpd17042[c] + 0.00309647 cpd15560[c] + 0.135407 cpd00038[c] + 0.00309647 cpd00048[c] + 0.0841036 cpd00052[c] + 0.00309647 cpd00201[c] + 0.00309647 cpd00118[c] + 0.00309647 cpd00264[c] + 0.00309647 cpd00015[c] + 0.00309647 cpd00345[c] + 0.154519 cpd00066[c] + 0.00309647 cpd00016[c] + 0.0792636 cpd00119[c] + 0.219088 cpd00053[c] + 0.200831 cpd00132[c] + 0.427934 cpd00035[c] + 0.00309647 cpd00058[c] + 0.00309647 cpd00087[c] + 0.246697 cpd00051[c] + 0.00309647 cpd00220[c] + 0.375389 cpd00107[c] + 0.0160207 cpd00357[c] + 0.184355 cpd00129[c] + 0.120677 cpd00069[c] + 0.241799 cpd00322[c] + 0.285438 cpd00039[c] + 0.0160207 cpd00115[c] + 0.211073 cpd00161[c] + 0.0908319 cpd00062[c] + cpd17043[c] + 0.0472019 cpd00065[c] + 0.352233 cpd00156[c] + cpd17041[c] + 0.00309647 cpd15500[c] + 0.00309647 cpd15352[c] + 0.00309647 cpd10516[c] + 0.0160207 cpd00241[c] + 0.00309647 cpd00254[c] + 0.00309647 cpd00205[c] + 0.00309647 cpd00099[c] + 0.00309647 cpd00149[c] + 0.00309647 cpd00166[c] + 0.0250106 cpd02229[c] + 0.0250106 cpd15665[c] + 0.00309647 cpd10515[c] + 0.00309647 cpd00028[c] + 0.010648 cpd15696[c] + 0.00309647 cpd00030[c] + 0.010648 cpd15533[c] + 0.010648 cpd15794[c] + 0.010648 cpd15723[c] + 0.010648 cpd15695[c] + 0.010648 cpd15540[c] + 0.00309647 cpd00063[c] + 0.010648 cpd15722[c] + 0.00309647 cpd00034[c] + 0.010648 cpd15793[c] + 0.0250106 cpd15432[c] + 0.010648 cpd15795[c] + 0.00309647 cpd00557[c] -> 0.484601 cpd00012[c] + 40 cpd00067[c] + 40 cpd00008[c] + 39.9969 cpd00009[c] + 0.00309647 cpd12370[c] + 0.00309647 cpd03422[c] + 0.00309647 cpd01997[c] + 0.0250106 cpd15666[c] + cpd11416[c]

	40.1102 ATP[c] + 0.50987 Glycine[c] + 0.00309647 Glutathione[c] + 0.00309647 Thiamine diphosphate[c] + 35.5403 H2O[c] + 0.200831 L-Aspartate[c] + 0.00309647 Acylcarnitine[c] + 0.127801 L-Methionine[c] + 0.179456 L-Serine[c] + 0.00309647 Coenzyme A[c] + 0.219088 Glutamate[c] + 0.0761465 L-Cysteine[c] + 0.00309647 NAD[c] + 0.0160207 Deoxycytidine 5'-triphosphate[c] + 0.00309647 NADP[c] + 0.00309647 ACP[c] + DNA replication[c] + 0.00309647 Ubiquinone-8[c] + 0.135407 GTP[c] + 0.00309647 Sulfate[c] + 0.0841036 CTP[c] + 0.00309647 10-formyl-THF mono-L-glutamate[c] + 0.00309647 Putrescine[c] + 0.00309647 Spermidine[c] + 0.00309647 Flavin adenine dinucleotide[c] + 0.00309647 5-Methyltetrahydrofolate[c] + 0.154519 L-Phenylalanine[c] + 0.00309647 Pyridoxal phosphate[c] + 0.0792636 L-Histidine[c] + 0.219088 L-Glutamine[c] + 0.200831 L-Asparagine[c] + 0.427934 L-Alanine[c] + 0.00309647 Cu(II)[c] + 0.00309647 Tetrahydrofolate[c] + 0.246697 L-Arginine[c] + 0.00309647 Riboflavin[c] + 0.375389 L-Leucine[c] + 0.0160207 Deoxythymidine triphosphate[c] + 0.184355 L-Proline[c] + 0.120677 L-Tyrosine[c] + 0.241799 L-Isoleucine[c] + 0.285438 L-Lysine[c] + 0.0160207 dATP[c] + 0.211073 L-Threonine [c] + 0.0908319 UTP [c] + RNA transcription[c] + 0.0472019 L-Tryptophan [c] + 0.352233 L-Valine [c] + Protein biosynthesis[c] + 0.00309647 Menaquinone 8[c] + 0.00309647 2-Demethylmenaquinone 8[c] + 0.00309647 Fe(III) [c] + 0.0160207 dGTP[c] + 0.00309647 Mg(2+)[c] + 0.00309647 K(+)[c] + 0.00309647 Chloride ion[c] + 0.00309647 Co(II)[c] + 0.00309647 Calomide[c] + 0.0250106 Calomide[c] + 0.0250106 Peptidoglycan polymer (n subunits)[c] + 0.00309647 Fe(II)[c] + 0.00309647 Heme[c] + 0.010648 Dianteisoheptadecanoylphosphatidylethanolamine[c] + 0.00309647 Mn(II)[c] + 0.010648 Phosphatidylethanolamine dioctadecanoyl[c] + 0.010648 Isoheptadecanoylcardiolipin[c] + 0.010648 Dianteisoheptadecanoylphosphatidylglycerol[c] + 0.010648 Diisoheptadecanoylphosphatidylethanolamine[c] + 0.010648 Phosphatidylglycerol dioctadecanoyl[c] + 0.00309647 Ca(2+)[c] + 0.010648 Diisoheptadecanoylphosphatidylglycerol[c] + 0.00309647 Zn(II)[c] + 0.010648 Stearoylcardiolipin[c] + 0.0250106 Core oligosaccharide lipid A[c] + 0.010648 Anteisoheptadecanoylcardiolipin[c] + 0.00309647 Siroheme[c] -> 0.484601 Diphosphoric acid[c] + 40 H(+)[c] + 40 ADP[c] + 39.9969 Phosphate[c] + 0.00309647 apo-ACP[c] + 0.00309647 Cobinamide[c] + 0.00309647 Dimethylbenzimidazole[c] + 0.0250106 Peptidoglycan polymer (n-1 subunits)[c] + Biomass[c]
**S4**	40.1102 cpd00002[c] + 0.50987 cpd00033[c] + 0.00309647 cpd00042[c] + 0.00309647 cpd00056[c] + 35.5403 cpd00001[c] + 0.200831 cpd00041[c] + 0.00309647 cpd00017[c] + 0.127801 cpd00060[c] + 0.00309647 cpd00010[c] + 0.219088 cpd00023[c] + 0.0761465 cpd00084[c] + 0.0160207 cpd00356[c] + 0.00309647 cpd00003[c] + 0.285438 cpd00039[c] + 0.00309647 cpd11493[c] + cpd17042[c] + 0.00309647 cpd15560[c] + 0.179456 cpd00054[c] + 0.135407 cpd00038[c] + 0.00309647 cpd00048[c] + 0.0841036 cpd00052[c] + 0.00309647 cpd00201[c] + 0.00309647 cpd00006[c] + 0.00309647 cpd00015[c] + 0.00309647 cpd00118[c] + 0.00309647 cpd00345[c] + 0.154519 cpd00066[c] + 0.427934 cpd00035[c] + 0.00309647 cpd00016[c] + 0.0792636 cpd00119[c] + 0.219088 cpd00053[c] + 0.200831 cpd00132[c] + 0.00309647 cpd15500[c] + 0.00309647 cpd15352[c] + 0.010648 cpd15533[c] + 0.00309647 cpd00058[c] + 0.00309647 cpd00087[c] + 0.246697 cpd00051[c] + 0.00309647 cpd00220[c] + 0.0160207 cpd00357[c] + 0.184355 cpd00129[c] + 0.120677 cpd00069[c] + 0.241799 cpd00322[c] + 0.0160207 cpd00115[c] + 0.211073 cpd00161[c] + 0.0908319 cpd00062[c] + cpd17043[c] + 0.00309647 cpd00264[c] + 0.0472019 cpd00065[c] + 0.352233 cpd00156[c] + 0.375389 cpd00107[c] +

	cpd17041[c] + 0.0160207 cpd00241[c] + 0.00309647 cpd00254[c] + 0.00309647 cpd00205[c] + 0.00309647 cpd00099[c] + 0.00309647 cpd00166[c] + 0.00309647 cpd10516[c] + 0.00309647 cpd00149[c] + 0.010648 cpd15696[c] + 0.0250106 cpd02229[c] + 0.0250106 cpd15665[c] + 0.010648 cpd15695[c] + 0.00309647 cpd10515[c] + 0.00309647 cpd00028[c] + 0.010648 cpd15793[c] + 0.00309647 cpd00030[c] + 0.00309647 cpd00063[c] + 0.0250106 cpd15432[c] + 0.010648 cpd15540[c] + 0.00309647 cpd00034[c] + 0.010648 cpd15723[c] + 0.00309647 cpd00557[c] + 0.010648 cpd15794[c] + 0.010648 cpd15795[c] + 0.010648 cpd15722[c] <=> 0.484601 cpd00012[c] + 40 cpd00008[c] + 39.9969 cpd00009[c] + 0.00309647 cpd03422[c] + 0.00309647 cpd01997[c] + 0.0250106 cpd15666[c] + cpd11416[c] + 0.00309647 cpd12370[c]
	40.1102 ATP[c] + 0.50987 Glycine[c] + 0.00309647 Glutathione[c] + 0.00309647 Thiamine diphosphate[c] + 35.5403 H2O[c] + 0.200831 L-Aspartate[c] + 0.00309647 Acylcarnitine[c] + 0.127801 L-Methionine[c] + 0.00309647 Coenzyme A[c] + 0.219088 Glutamate[c] + 0.0761465 L-Cysteine[c] + 0.0160207 Deoxycytidine 5'-triphosphate[c] + 0.00309647 NAD[c] + 0.285438 L-Lysine[c] + 0.00309647 ACP[c] + DNA replication[c] + 0.00309647 Ubiquinone-8[c] + 0.179456 L-Serine[c] + 0.135407 GTP[c] + 0.00309647 Sulfate[c] + 0.0841036 CTP[c] + 0.00309647 10-formyl-THF mono-L-glutamate[c] + 0.00309647 NADP[c] + 0.00309647 Flavin adenine dinucleotide[c] + 0.00309647 Putrescine[c] + 0.00309647 5-Methyltetrahydrofolate[c] + 0.154519 L-Phenylalanine[c] + 0.427934 L-Alanine[c] + 0.00309647 Pyridoxal phosphate[c] + 0.0792636 L-Histidine[c] + 0.219088 L-Glutamine[c] + 0.200831 L-Asparagine[c] + 0.00309647 Menaquinone 8[c] + 0.00309647 2-Demethylmenaquinone 8[c] + 0.010648 Phosphatidylethanolamine dioctadecanoyl[c] + 0.00309647 Cu(II)[c] + 0.00309647 Tetrahydrofolate[c] + 0.246697 L-Arginine[c] + 0.00309647 Riboflavin[c] + 0.0160207 Deoxythymidine triphosphate[c] + 0.184355 L-Proline[c] + 0.120677 L-Tyrosine[c] + 0.241799 L-Isoleucine[c] + 0.0160207 dATP[c] + 0.211073 L-Threonine [c] + 0.0908319 UTP [c] + RNA transcription[c] + 0.00309647 Spermidine[c] + 0.0472019 L-Tryptophan [c] + 0.352233 L-Valine [c] + 0.375389 L-Leucine[c] + Protein biosynthesis[c] + 0.0160207 dGTP[c] + 0.00309647 Mg(2+)[c] + 0.00309647 K(+)[c] + 0.00309647 Chloride ion[c] + 0.00309647 Calomide[c] + 0.00309647 Fe(III) [c] + 0.00309647 Co(II)[c] + 0.010648 Dianteisoheptadecanoylphosphatidylethanolamine[c] + 0.0250106 Calomide[c] + 0.0250106 Peptidoglycan polymer (n subunits)[c] + 0.010648 Diisoheptadecanoylphosphatidylethanolamine[c] + 0.00309647 Fe(II)[c] + 0.00309647 Heme[c] + 0.010648 Stearoylcardiolipin[c] + 0.00309647 Mn(II)[c] + 0.00309647 Ca(2+)[c] + 0.0250106 Core oligosaccharide lipid A[c] + 0.010648 Phosphatidylglycerol dioctadecanoyl[c] + 0.00309647 Zn(II)[c] + 0.010648 Dianteisoheptadecanoylphosphatidylglycerol[c] + 0.00309647 Siroheme[c] + 0.010648 Isoheptadecanoylcardiolipin[c] + 0.010648 Anteisoheptadecanoylcardiolipin[c] + 0.010648 Diisoheptadecanoylphosphatidylglycerol[c] <=> 0.484601 Diphosphoric acid[c] + 40 ADP[c] + 39.9969 Phosphate[c] + 0.00309647 Cobinamide[c] + 0.00309647 Dimethylbenzimidazole[c] + 0.0250106 Peptidoglycan polymer (n-1 subunits)[c] + Biomass[c] + 0.00309647 apo-ACP[c]
