# Supplementary material for: Potentiators empower synthetic microbiomes as silent guardians against co-contamination
Source: Nat Commun. 2025 Dec 31;17:1185. doi: 10.1038/s41467-025-67953-5 (PMC12858907; doi:10.1038/s41467-025-67953-5)
Supplement: Supplementary file 6 — Supplementary Data 4 [file 41467_2025_67953_MOESM6_ESM.pdf]

Supplementary Data 4 Flux balance analysis illustrating interspecies metabolic exchange under the objective of equalizing biomass for each species in the community. Using the SuperCC modeling framework, the maximum biomass was simulated for six-strain combinations under two nutritional scenarios: (1) MM supplemented with 100 mmol/gDW glucose (single carbon source, C = 1); and (2) MM with 25 mmol/gDW each of glucose, citrate, acetate, and fumarate (quadruple carbon sources, C = 4). Substances with a light blue background represent metabolites unique to the C = 4 condition.

**C = 1**					
**Secretion**	**Compounds**	**Formula**	**ModelSEED ID**	**Absorption**	**Category**
**A1**	L-Lysine	C6H15N2O2	cpd00039	P1, C2, L3	Amino Acids and Derivatives
	Octadecanoic acid	C18H35O2	cpd01080	P1, C2, S4	Organic Acids
	(R)-3-Hydroxybutanoate	C4H7O3	cpd00797	C2	Organic Acids
	D-Glucosamine	C6H14NO5	cpd00276	P1	Saccharides and Derivatives
	Succinate	C4H4O4	cpd00036	C2, S4	Organic Acids
	L-Glutamate	C5H8NO4	cpd00023	C2, L3, S4	Amino Acids and Derivatives
	Xanthinethine	C5H4N4O2	cpd00309	P1, C2	Nucleosides/Nucleotides and Derivatives
**L3**	D-Alanine	C3H7NO2	cpd00117	A1, C2	Amino Acids and Derivatives
	Glycine	C2H5NO2	cpd00033	A1, P1	Amino Acids and Derivatives
	Urea	CH4N2O	cpd00073	C2	Organic Acids
	Ornithine	C5H13N2O2	cpd00064	P1	Nucleosides/Nucleotides and Derivatives
	L-Proline	C5H9NO2	cpd00129	A1, P1	Amino Acids and Derivatives
	L-Serine	C3H7NO3	cpd00054	A1, C2	Amino Acids and Derivatives
	Glycerol	C3H8O3	cpd00100	P1	Saccharides and Derivatives
	Acetate	C2H3O2	cpd00029	A1, P1, S4	Organic Acids
**B4**	Octadecanoic acid	C18H35O2	cpd01080	P1, C2, S4	Organic Acids
	L-Glutamate	C5H8NO4	cpd00023	C2, L3, S4	Amino Acids and Derivatives
	Uridine	C9H12N2O6	cpd00249	P1	Nucleosides/Nucleotides and Derivatives
	Inosine	C10H12N4O5	cpd00246	P1	Nucleosides/Nucleotides and Derivatives
	Acetate	C2H3O2	cpd00029	A1, P1, S4	Organic Acids

	2-Oxoglutarate	$C_5H_4O_5$	cpd00024	P1, C2	Organic Acids
	L-Lysine	$C_6H_{15}N_2O_2$	cpd00039	P1, C2, L3	Amino Acids and Derivatives
**P1**	L-Tyrosine	$C_9H_{11}NO_3$	cpd00069	C2	Amino Acids and Derivatives
	L-Phenylalanine	$C_9H_{11}NO_2$	cpd00066	A1, C2	Amino Acids and Derivatives
	Uracil	$C_4H_4N_2O_2$	cpd00092	A1, C2, L3	Nucleosides/Nucleotides and Derivatives
	Myristic acid	$C_{14}H_{27}O_2$	cpd03847	L3, B4, S4	Organic Acids
	Hypoxanthine	$C_5H_4N_4O$	cpd00226	A1, L3, S4	Nucleosides/Nucleotides and Derivatives
	N-Acetyl-D-glucosamine	$C_8H_{15}NO_6$	cpd00122	S4	Saccharides and Derivatives
	Cytidine	$C_9H_{13}N_3O_5$	cpd00367	B4	Nucleosides/Nucleotides and Derivatives
	L-Arginine	$C_6H_{15}N_4O_2$	cpd00051	L3	Amino Acids and Derivatives
	D-Alanine	$C_3H_7NO_2$	cpd00117	A1, C2	Amino Acids and Derivatives
	L-Serine	$C_3H_7NO_3$	cpd00054	A1, C2	Amino Acids and Derivatives
	D-Trehalose	$C_{12}H_{22}O_{11}$	cpd00794	S4	Saccharides and Derivatives
	Adenosine	$C_{10}H_{13}N_5O_4$	cpd00182	B4	Nucleosides/Nucleotides and Derivatives
	L-Glutamate	$C_5H_8NO_4$	cpd00023	C2, L3, S4	Amino Acids and Derivatives
	Succinate	$C_4H_4O_4$	cpd00036	C2, S4	Organic Acids
	D-Mannose	$C_6H_{12}O_6$	cpd00138	A1, C2	Saccharides and Derivatives
**C2**	Thiamin	$C_{12}H_{17}N_4OS$	cpd00305	A1, P1, L3	Vitamins
	L-Tryptophan	$C_{11}H_{12}N_2O_2$	cpd00065	P1, B4	Amino Acids and Derivatives
	L-Proline	$C_5H_9NO_2$	cpd00129	A1, P1	Amino Acids and Derivatives
	Fumarate	$C_4H_2O_4$	cpd00106	A1, P1	Saccharides and Derivatives
	Acetate	$C_2H_3O_2$	cpd00029	A1, P1, S4	Organic Acids
	D-Fructose	$C_6H_{12}O_6$	cpd00082	A1, P1	Saccharides and Derivatives
	Glycine	$C_2H_5NO_2$	cpd00033	A1, P1	Amino Acids and Derivatives
	D-Glucosamine	$C_6H_{14}NO_5$	cpd00276	P1	Saccharides and Derivatives
	Hypoxanthine	$C_5H_4N_4O$	cpd00226	A1, L3, S4	Nucleosides/Nucleotides and Derivatives
**S4**	Nicotinamide ribonucleotide	$C_{11}H_{14}N_2O_8P$	cpd00355	P1	Vitamins

	Compounds	Formula	ModelSEED ID	Absorption	Category
	Palmitate	C16H31O2	cpd00214	A1	Organic Acids
	(R)-3-Hydroxybutanoate	C4H7O3	cpd00797	C2	Organic Acids
	D-Glucosamine	C6H14NO5	cpd00276	P1	Saccharides and Derivatives
	D-Mannose	C6H12O6	cpd00138	A1, C2	Saccharides and Derivatives
	Fumarate	C4H2O4	cpd00106	A1, P1	Saccharides and Derivatives
**C = 4**					
**Secretion**	**Compounds**	**Formula**	**ModelSEED ID**	**Absorption**	**Category**
**A1**	Octadecanoic acid	C18H35O2	cpd01080	P1, B4, S4	Organic Acids
	Succinate	C4H4O4	cpd00036	C2, S4	Organic Acids
	Hypoxanthine	C5H4N4O	cpd00226	C2, L3, S4	Nucleosides/Nucleotides and Derivatives
	L-Lysine	C6H15N2O2	cpd00039	P1, C2, L3	Amino Acids and Derivatives
	D-Glucosamine	C6H14NO5	cpd00276	P1, S4	Saccharides and Derivatives
**B4**	Inosine	C10H12N4O5	cpd00246	P1	Nucleosides/Nucleotides and Derivatives
	Uridine	C9H12N2O6	cpd00249	P1	Nucleosides/Nucleotides and Derivatives
	Deoxyuridine	C9H12N2O5	cpd00412	P1	Nucleosides/Nucleotides and Derivatives
	L-Lysine	C6H15N2O2	cpd00039	P1, C2, L3	Amino Acids and Derivatives
	2-Oxoglutarate	C5H4O5	cpd00024	P1	Organic Acids
**C2**	Thiamin	C12H17N4OS	cpd00305	A1, P1, L3	Vitamins
	4-Aminobutanoic acid	C4H9NO2	cpd00281	L3	Amino Acids and Derivatives
	L-Tryptophan	C11H12N2O2	cpd00065	P1, B4	Amino Acids and Derivatives
	Uracil	C4H4N2O2	cpd00092	A1, L3	Nucleosides/Nucleotides and Derivatives
	Xanthinethine	C5H4N4O2	cpd00309	A1, P1	Nucleosides/Nucleotides and Derivatives
	(R)-3-Hydroxybutanoate	C4H7O3	cpd00797	A1	Organic Acids
	L-Proline	C5H9NO2	cpd00129	A1, P1	Amino Acids and Derivatives
	Glycine	C2H5NO2	cpd00033	A1, P1	Amino Acids and Derivatives
	D-Glucosamine	C6H14NO5	cpd00276	P1, S4	Saccharides and Derivatives
**L3**	Cytosine	C4H5N3O	cpd00307	C2	Nucleosides/Nucleotides and Derivatives

	L-Proline	C5H9NO2	cpd00129	A1, P1	Amino Acids and Derivatives
	Glycine	C2H5NO2	cpd00033	A1, P1	Amino Acids and Derivatives
	D-Alanine	C3H7NO2	cpd00117	A1, C2	Amino Acids and Derivatives
	L-Serine	C3H7NO3	cpd00054	A1, C2	Amino Acids and Derivatives
	Urea	CH4N2O	cpd00073	P1, C2	Organic Acids
	Ornithine	C5H13N2O2	cpd00064	P1	Nucleosides/Nucleotides and Derivatives
**P1**	Adenosine	C10H13N5O4	cpd00182	B4	Nucleosides/Nucleotides and Derivatives
	L-Tyrosine	C9H11NO3	cpd00069	C2	Amino Acids and Derivatives
	Cytidine	C9H13N3O5	cpd00367	B4	Nucleosides/Nucleotides and Derivatives
	Uracil	C4H4N2O2	cpd00092	A1, L3	Nucleosides/Nucleotides and Derivatives
	L-Phenylalanine	C9H11NO2	cpd00066	A1, C2, S4	Amino Acids and Derivatives
	Myristic acid	C14H27O2	cpd03847	L3, B4, S4	Organic Acids
	Hypoxanthine	C5H4N4O	cpd00226	C2, L3, S4	Nucleosides/Nucleotides and Derivatives
	Glycerol	C3H8O3	cpd00100	L3	Saccharides and Derivatives
	D-Fructose	C6H12O6	cpd00082	A1, C2	Saccharides and Derivatives
	Succinate	C4H4O4	cpd00036	C2, S4	Organic Acids
	Deoxycytidine	C9H13N3O4	cpd00654	B4	Nucleosides/Nucleotides and Derivatives
	D-Trehalose	C12H22O11	cpd00794	S4	Saccharides and Derivatives
	D-Alanine	C3H7NO2	cpd00117	A1, C2	Amino Acids and Derivatives
	L-Serine	C3H7NO3	cpd00054	C2	Amino Acids and Derivatives
	L-Glutamate	C5H8NO4	cpd00023	A1, C2, L3, B4, S4	Amino Acids and Derivatives
	L-Arginine	C6H15N4O2	cpd00051	L3	Amino Acids and Derivatives
	D-Mannose	C6H12O6	cpd00138	A1, C2	Saccharides and Derivatives
**S4**	Nicotinamide ribonucleotide	C11H14N2O8P	cpd00355	P1	Vitamins
	Palmitate	C16H31O2	cpd00214	A1	Organic Acids
	(R)-3-Hydroxybutanoate	C4H7O3	cpd00797	A1	Organic Acids
	N-Acetyl-D-glucosamine	C8H15NO6	cpd00122	P1	Saccharides and Derivatives

	D-Mannose	$C_6H_{12}O_6$	cpd00138	A1, C2	Saccharides and Derivatives
