# Supplementary material for: Potentiators empower synthetic microbiomes as silent guardians against co-contamination
Source: Nat Commun. 2025 Dec 31;17:1185. doi: 10.1038/s41467-025-67953-5 (PMC12858907; doi:10.1038/s41467-025-67953-5)
Supplement: Supplementary file 8 — Reporting Summary [file 41467_2025_67953_MOESM8_ESM.pdf]

Corresponding author(s): Rongliang Qiu
Last updated by author(s): Nov 20, 2025

Reporting Summary

Nature Portfolio wishes to improve the reproducibility of the work that we publish. This form provides structure for consistency and transparency in reporting. For further information on Nature Portfolio policies, see our Editorial Policies and the Editorial Policy Checklist.

Statistics

For all statistical analyses, confirm that the following items are present in the figure legend, table legend, main text, or Methods section.

n/a	Confirmed	
☐	☒	The exact sample size (*n*) for each experimental group/condition, given as a discrete number and unit of measurement
☐	☒	A statement on whether measurements were taken from distinct samples or whether the same sample was measured repeatedly
☐	☒	The statistical test(s) used AND whether they are one- or two-sided<br>*Only common tests should be described solely by name; describe more complex techniques in the Methods section.*
☒	☐	A description of all covariates tested
☐	☒	A description of any assumptions or corrections, such as tests of normality and adjustment for multiple comparisons
☐	☒	A full description of the statistical parameters including central tendency (e.g. means) or other basic estimates (e.g. regression coefficient) AND variation (e.g. standard deviation) or associated estimates of uncertainty (e.g. confidence intervals)
☐	☒	For null hypothesis testing, the test statistic (e.g. *F*, *t*, *r*) with confidence intervals, effect sizes, degrees of freedom and *P* value noted<br>*Give P values as exact values whenever suitable.*
☒	☐	For Bayesian analysis, information on the choice of priors and Markov chain Monte Carlo settings
☒	☐	For hierarchical and complex designs, identification of the appropriate level for tests and full reporting of outcomes
☒	☐	Estimates of effect sizes (e.g. Cohen's *d*, Pearson's *r*), indicating how they were calculated

Our web collection on statistics for biologists contains articles on many of the points above.

Software and code

Policy information about availability of computer code

Data collection	Microbiome sequencing data were dereplicated and processed using the DADA2 algorithm in QIIME 2 to identify insertions, deletions, and substitutions. Phylogenetic classification of each 16S rRNA gene sequence, referred to as amplicon sequence variants (ASVs), was performed using the SILVA SSU132 16S rRNA database with a confidence threshold of 70%.<br>Whole-genome sequencing data were quality-filtered using Trimmomatic (v0.36), and genome assembly was performed with Unicycler using default parameters, which yielded optimal results. Gene models were predicted using GeneMark and functionally annotated by BLASTp searches against the NCBI non-redundant (NR) database, SwissProt (http://uniprot.org), KEGG (http://www.genome.jp/kegg/), and COG (http://www.ncbi.nlm.nih.gov/COG). Transfer RNA (tRNA) genes were identified using tRNAscan-SE (v1.23), and ribosomal RNA (rRNA) genes were determined using RNAmmer (v1.2).<br>Metabolomic data of the microbiome were processed using Progenesis QI software for baseline filtering, peak identification and integration, retention time correction, and alignment. The resulting data matrix (sample names, m/z, RT, and intensities) was exported, and metabolites were annotated via HMDB, METLIN, and MJDB (Majorbio). Preprocessed data were further analyzed on the Majorbio Cloud Platform, including retention of variables present in ≥80% of samples, imputation of missing values (using the minimum value), and sum normalization. Gen5 3.11 was used to collect OD600 data generated by the microplate reader. Agilent OpenLab CDS 1.07 was used to collect data from HPLC, and Agilent MassHunter B.10.1 was used to acquire data from LC/MS.
Data analysis	Rarefaction analysis was performed using Mothur (v1.21.1) to estimate the Shannon index. β-diversity was calculated with the vegan package (v2.5-7). Microbiome functional profiles from metagenomic data were ordinated by PCA using unweighted UniFrac distances with KEGG modules. Marker ASVs discriminating treatment times were identified using a random forest approach with the randomForest package (v4.6-14) in R (v4.0.3).Data visualization and statistical analyses were conducted in GraphPad Prism 8.0 (GraphPad Software, La Jolla, CA, USA). Microbiome graphs were generated using ggplot2 (v3.3.0), pheatmap (v1.0.12), and VennDiagram (v1.6.20).<br>Draft metabolic models were initially constructed with Model SEED and further curated using COBRA Toolbox 3.0 in MATLAB (R2019a). Details

of the newly developed metabolic modeling pipeline, SuperCC, for optimal community combination analysis are available at https://github.com/ruanzhepu/superCC.

For manuscripts utilizing custom algorithms or software that are central to the research but not yet described in published literature, software must be made available to editors and reviewers. We strongly encourage code deposition in a community repository (e.g. GitHub). See the Nature Portfolio guidelines for submitting code & software for further information.

Data

Policy information about availability of data

All manuscripts must include a data availability statement. This statement should provide the following information, where applicable:

- Accession codes, unique identifiers, or web links for publicly available datasets
- A description of any restrictions on data availability
- For clinical datasets or third party data, please ensure that the statement adheres to our policy

All amplicon sequencing data have been deposited in the NCBI Sequence Read Archive (SRA) under the accession number PRJNA1153784 CODE [https://www.ncbi.nlm.nih.gov/bioproject/1153784]. The GenBank accession numbers for genome sequences of the isolates are CP190002-CP190008. The metabolomics data have been deposited in the MetaboLights database under the accession number MTBLS12809 CODE [https://www.ebi.ac.uk/metabolights/MTBLS12809]. The data that support this study are available within the article and its Supplementary Information files. Source data are provided with this paper. The optimization models, media, and SuperCC function used in MATLAB are available at https://github.com/ruanzhepu/superCC.git.

Research involving human participants, their data, or biological material

Policy information about studies with human participants or human data. See also policy information about sex, gender (identity/presentation), and sexual orientation and race, ethnicity and racism.

Reporting on sex and gender	Not applicable. The study does not involve any human participants or human data.
Reporting on race, ethnicity, or other socially relevant groupings	Not applicable. The study does not involve any human participants or human data.
Population characteristics	Not applicable. The study does not involve any human participants or human data.
Recruitment	Not applicable. The study does not involve any human participants or human data.
Ethics oversight	Not applicable. The study does not involve any human participants or human data.

Note that full information on the approval of the study protocol must also be provided in the manuscript.

Field-specific reporting

Please select the one below that is the best fit for your research. If you are not sure, read the appropriate sections before making your selection.

☒ Life sciences ☐ Behavioural & social sciences ☐ Ecological, evolutionary & environmental sciences

For a reference copy of the document with all sections, see nature.com/documents/nr-reporting-summary-flat.pdf

Life sciences study design

All studies must disclose on these points even when the disclosure is negative.

Sample size	No statistical methods were used to predetermine sample size. All experiments were performed with a minimum of three biological replicates (n≥3) unless otherwise stated in the figure legends.
Data exclusions	The study retained all collected data without exclusion.
Replication	All the samples were performed in triplicate or higher, as noted in the text.
Randomization	Randomization was not applicable in this study as all bacterial strains received identical treatment protocols.
Blinding	Blinding was not necessary as strict inclusion/exclusion criteria were set ensuring that objectivity was maintained in article selection.

Reporting for specific materials, systems and methods

We require information from authors about some types of materials, experimental systems and methods used in many studies. Here, indicate whether each material, system or method listed is relevant to your study. If you are not sure if a list item applies to your research, read the appropriate section before selecting a response.

Materials & experimental systems

n/a	Involved in the study
☒	☐ Antibodies
☒	☐ Eukaryotic cell lines
☒	☐ Palaeontology and archaeology
☒	☐ Animals and other organisms
☒	☐ Clinical data
☒	☐ Dual use research of concern
☒	☐ Plants

Methods

n/a	Involved in the study
☒	☐ ChIP-seq
☒	☐ Flow cytometry
☒	☐ MRI-based neuroimaging

Plants

Seed stocks	No seed stocks of plants were involved in this study.
Novel plant genotypes	No plants with novel genotypes were involved in this study.
Authentication	No authentication procedures were involved in this study.
